# Evolocumab loaded Bio-Liposomes for efficient atherosclerosis therapy

**DOI:** 10.1186/s12951-023-01904-4

**Published:** 2023-05-19

**Authors:** Zhenxian Li, Haimei Zhu, Hao Liu, Dayue Liu, Jianhe Liu, Jiazheng Jiang, Yi Zhang, Zhang Qin, Yijia Xu, Yuan Peng, Bin Liu, Yun Long

**Affiliations:** 1 Department of Cardiology, The First Hospital of Hunan University of Chinese Medicine, Branch of National Clinical Research Center for Chinese Medicine Cardiology, Changsha, 410007 China; 2 College of Biology, Hunan University, Changsha, 410082 China; 3 Department of Rehabilitation, The Second Xiangya Hospital, Central South University, Changsha, 410011 China; 4 Department of Physiology and Pathophysiology, NHC Key Laboratory of Metabolic Cardiovascular Diseases Research, School of Basic Medical Sciences, Ningxia Medical University, Yinchuan, 750004 China; 5 Department of Pain, The First Hospital of Hunan University of Chinese Medicine, Changsha, 410007 China

**Keywords:** Atherosclerosis, Evolocumab, Proprotein convertase subtilisin/Kexin type 9, Liposome, Phenotypic transition

## Abstract

PCSK9, which is closely related to atherosclerosis, is significantly expressed in vascular smooth muscle cells (VSMCs). Moreover, Proprotein Convertase Subtilisin/Kexin type 9 (PCSK9) mediated phenotypic transformation, abnormal proliferation, and migration of VSMCs play key roles in accelerating atherosclerosis. In this study, by utilizing the significant advantages of nano-materials, a biomimetic nanoliposome loading with Evolocumab (Evol), a PCSK9 inhibitor, was designed to alleviate atherosclerosis. In vitro results showed that (Lipo + M)@E NPs up-regulated the levels of α-SMA and Vimentin, while inhibiting the expression of OPN, which finally result in the inhibition of the phenotypic transition, excessive proliferation, and migration of VSMCs. In addition, the long circulation, excellent targeting, and accumulation performance of (Lipo + M)@E NPs significantly decreased the expression of PCSK9 in serum and VSMCs within the plaque of ApoE^−/−^ mice.

**Supplementary Information:**

The online version contains supplementary material available at 10.1186/s12951-023-01904-4.

## Introduction

It is well-known that changes in the structure and function of VSMCs are closely related to the occurrence of atherosclerosis [1]. Frismantiene et al*.* found that VSMCs-derived cells accounted for more than 70% of atherosclerotic plaque cells [2]. Under normal circumstances, proliferation, and migration of VSMCs can repair vascular injury. However, inflammatory stimuli and oxidative stress can cause phenotypic transition of VSMCs from contractile to synthetic type. The abnormal proliferation and migration of synthetic VSMCs can accelerate atherosclerosis development by promoting plaque formation [3]. Experimental studies have indicated that early intervention in the phenotypic transformation of VSMCs can improve the progression and prognosis of atherosclerosis by inhibiting PCSK9 [4]. Further study indicated that PCSK9 could directly regulate the levels of alpha-smooth muscle actin (α-SMA), osteopontin (OPN), and Vimentin, key molecules mediating phenotypic transformation of VSMCs [5]. Evolocumab (Evol), a proprotein convertase subtilisin/Kexin type 9 (PCSK9) inhibitor, has attracted widespread attention in atherosclerosis therapy due to its function of lowering LDL-C [6]. However, how to reduce the dosage and enhance the targeting without affecting the efficacy is the key problem of using this drug in the treatment of atherosclerosis.

The appearance of various nano-materials brings hope for solving this problem [7]. As a kind of liposome with a phospholipid bilayer biofilm structure, nano-liposome can improve bioavailability and maintain their original properties by embedding active substances. So far, nanoliposomes have been used as drug carriers to treat cardiac diseases such as arrhythmia and myocardial infarction, and achieved satisfactory efficacy [8, 9]. However, the liposomes entering into the blood circulation often face the challenge of various factors such as albumin and opsonin, the rupture of liposomes results in the leakage of encapsulated drugs before arriving at the destinations. More importantly, liposomes lacking complex antigens present on natural cell membranes could not be  actively home to the disease site. Recently, Polyethylene glycol (PEG) was reported to efficiently prolong the half-life in vivo and ensure sustained drug concentration with low immunogenicity [10]. In addition, macrophages membrane (Møm) camouflage has endowed nano-material long circulation time in vivo as proteins and polysaccharides on cells membrane surface can prevent NPs from being attacked by the immune system [11, 12]. Thus, the characteristic proteins on the macrophage membrane can target and permeate atherosclerotic plaque through the “homing effect” [13].

**Fig. 1:**
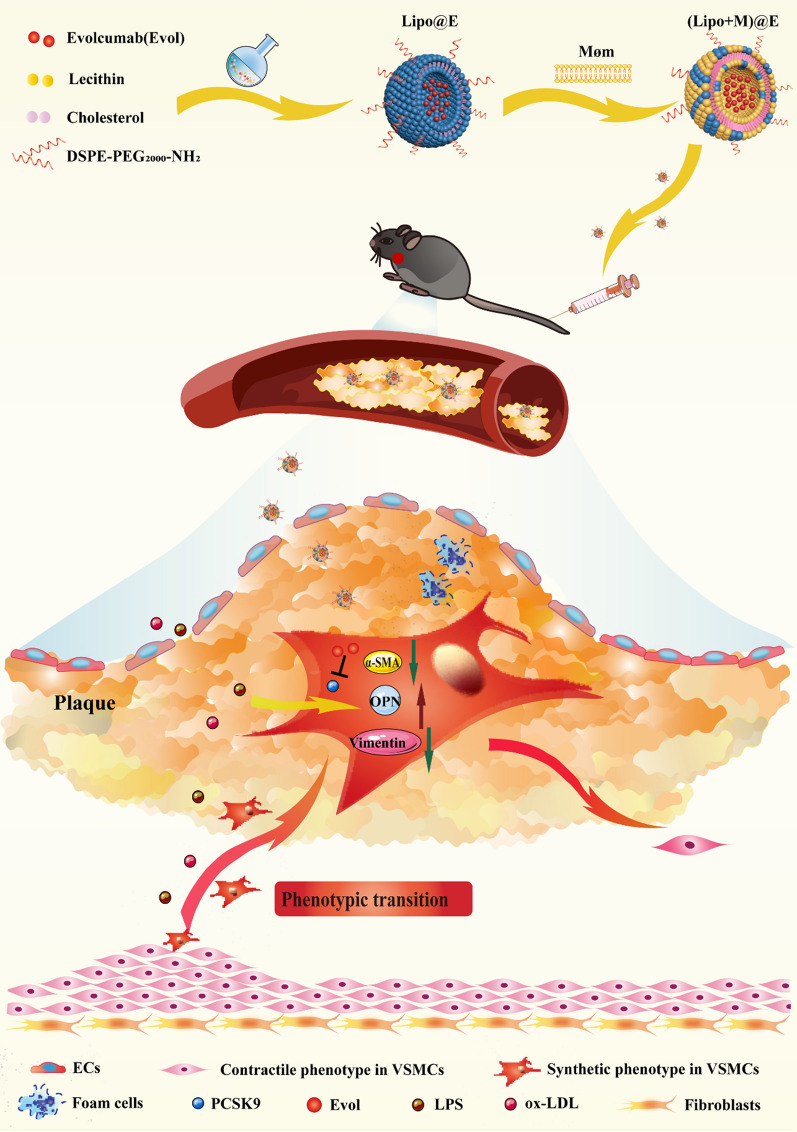
Synthesis process of (Lipo + M)@E NPs and the inhibiting mechanism of phenotypic transition of VSMCs by reducing PCSK9. First, the nanoliposomes were used to carry Evol with high drug-loading efficiency. PEG was used to modify the nanoliposomes. Møm was used to camouflage Lipo@E NPs to form (Lipo+M)@E NPs. After intravenous administration, (Lipo + M)@E NPs inhibited the expression of PCSK9, leading to the upregulation of the levels of α-SMA and Vimentin, while inhibiting the expression of OPN, which finally result in the inhibition of the phenotypic transition, excessive proliferation, and migration of VSMCs, thus effectively treating atherosclerosis.

Here, we constructed a biomimetic membrane-coated Evol delivery nanocomplexes for atherosclerosis therapy. First, the nanoliposomes were used to carry Evol with high drug loading efficiency. PEG was used to modify the nanoliposomes. Møm was used to camouflage Lipo@E NPs to form (Lipo + M)@E NPs. As a result, the new Evol-loaded nanocomplexes obtained long circulation life and high accumulate ability in atherosclerotic plaque. The downregulation of PCSK9 by (Lipo + M)@E NPs inhibited the proliferation and migration of VSMCs through inhibiting phenotype transformation in the plaque, further alleviating atherosclerotic lesion (Fig. 1).

## Experimental section

### Chemicals and reagents

Evolocumab was supplied by Amgen Manufacturing Limited (USA). l-Alpha-phosphatidyl choline (SPC) and cholesterol were purchased from Shanghai Ryon Biological Technology Co. Ltd (Shanghai, China). DSPE-PEG_2000_-NH_2_ was obtained from Pengsheng Company (Shanghai China). Lipopolysaccharide (LPS) and Oil Red O (ORO) were obtained from Sigma-Aldrich (MO, USA). BCA protein quantification kit was obtained from Yeasen Biotech. Co., Ltd. (Shanghai, China).

### Synthesis of nanoparticles

#### Preparation of Mø membrane

The RAW264.7 cell membrane was prepared using the Cell membrane protein extraction kit (Beyotime, China). In short, RAW264.7 cells were washed with cold PBS and centrifuged at 800 rpm for 10 min, then membrane protein extraction reagent A containing PMSF was added to the cell fragments and incubated for 30 min on the ice. After five cycles of freezing and thawing, the macrophages membrane (Møm) was collected through centrifugation.

#### Synthesis of Lipo@E and (Lipo + M)@E NPs

Lipo@E NPs composed of soybean lecithin/cholesterol/PEG/Evol = 2:1:0.5:0.2 (mass ratio) were prepared using the reverse evaporation method [14]. Soybean lecithin, cholesterol, and PEG were dissolved in the mixture containing 4 mL chloroform and 6 mL diethyl ether. The Evol solution was slowly added to the lipid solution with a syringe and ultrasonicated in a water bath for 5 min to form a W/O emulsion. The resulting emulsion was vaporized at 37 °C under reduced pressure in a rotary evaporator. 30 mL of PBS (pH 7.2) containing 2% Tween-80 was added to gelatinized solution. After removing the remaining organic solvent by distillation, the solution was rotated until the gel on the bottle wall fell off, and the emulsion nanoliposome suspension was obtained through a 0.45 μm microporous filtration membrane. Free Evol was removed by dialyzing Lipo@E NPs for 12 h. The harvested Lipo@E NPs were mixed with the Møm solution with a 5:1 phospholipid ratio and stirred at 37 °C for 2 h. Uniform (Lipo + M)@E NPs were obtained after sonication in a water bath and extruding through 0.22 μm polyethersulfone membrane.

### *Characterization*

Transmission electron microscope (TEM) was used to observe the topography characteristics of the nanoparticles. Dynamic light scattering (DLS, Nano ZS90 Zetasizer, Malvern) was used to detect the size and zeta potential of nanoparticles. DiL-labeled solution Lipo (red fluorescence) and DiO-labeled Møm solution (green fluorescence) were respectively prepared. Then, the two kinds of solutions were stirred in the dark for 2 h to prepare [Lipo + Mø] m NPs. The extent of membrane fusion was characterized under Nikon (CLSM, Olympus, FV1200, Japan). Membrane protein extraction kit was used to extract M@Lipo nanocomplex for protein extraction. Protein samples were separated and stained with Coomassie bright blue by 8% SDS-PAGE gel. In addition, CD11b, the specific surface marker of Møm was determined by western blot.

The release behaviors of Evol from Lipo@E NPs and (Lipo + M)@E NPs were investigated at pH 7.4. The formulations with equivalent loaded Evol dose (2 mg/mL, 1 mL) were placed in the dialysis bags (M.W. cutoff: 200 kDa), then immersed in 50 mL PBS (pH 7.4) and stirred in a 37 ℃ water bath. At each time point, 1 mL of removed solution was replaced by same volume of PBS solution. The content of Evol in PBS solution was detected using bicinchoninic acid (BCA) protein assay kit [15].

### *Cell uptake*

Vascular smooth muscle cell line (VSMC), mouse macrophage cell line (RAW264.7), rat cardiac myoblast cell line (H9C2), and human umbilical vein endothelial cell line (HUVEC) were purchased from the Cell Bank of the Chinese Academy of Sciences, and cultured in the DMEM (Gibco) with 10% FBS (Gibco) and 1% penicillin and streptomycin (Invitrogen). Activated VSMCs were prepared by treating with 100 ng/mL LPS for 24 h.

An in vitro binding experiment was performed to determine adhesion ability of M@Lipo NPs. Briefly, HUVECs were seeded in the 24-well plates (1 × 10^5^ cells/well). Then, 100 ng/mL LPS was added to the HUVECs with 70 − 80% confluence and continued to co-culture for 24 h. Then, DiD-labeled different NPs were added and cultured at 4 °C for 4 h. After that, the HUVECs were incubated with anti-ICAM-1(1:100, ab171123, Abcam). DAPI was used to stain the nucleus before observing under Nikon microscope (Ti-E + A1 MP, Japan).

The transwell chamber was adopted to simulate the atherosclerotic microenvironment. In short, HUVECs and VSMCs were separately cultured in the upper and lower chambers. Rhodamine B was used to label different NPs. After treatment with LPS (100 ng/mL) for 24 h, the different materials were added in the upper and lower chambers and the fluorescence images of cells incubating at 37 °C were captured 4 h later under confocal laser scanning microscopy (CLSM, Olympus, FV1200).

VSMCs seeded into 12-well plates with approximately 70% confluence were treated with DiL labeled M@Lipo NPs (corresponded to lecithin concentrations of 25, 50, 100, and 150 μg/mL) for 4 h. Meanwhile, the cells were stained with 1 μM Lyso Tracker Green DND-99 for 1 h. Hoechst 33342 was used to stain nuclei for 10 min, and the fluorescence images were captured under CLSM. In addition, VSMCs were preincubated with different uptake inhibitors for 1 h. Subsequently, DiL-labeled M@Lipo NPs were added and incubated for 4 h. CLSM was used to capture images after cells were stained by DAPI.

In vitro immune escape assay, RAW264.7 cells were incubated with DiL-labeled Lipo NPs and M@Lipo NPs (corresponded to lecithin concentrations of 25, 50, and 75 μg/mL) for 4 h. DAPI was used to stain nuclei before capturing under CLSM (Olympus, FV1200).

### The effect of (Lipo + M)@E NPs on the proliferation and migration of VSMCs

#### Viability assay of VSMCs

VSMCs were cultured in the 96-well plates (1 × 10^4^ cells/well) before the addition of LPS (100 ng/mL). After 24 h, Evol, Lipo@E NPs, and (Lipo + M)@E NPs were added and cultured for another 24 h. After adding MTT solution and incubating for 4 h, the absorbance values at 495 nm were measured.

#### Proliferation assay of VSMCs

VSMCs suspension was seeded in the 12-well plates (8 × 10^4^ cells/well). After treatment with LPS (100 ng/mL) for 24 h, different materials (corresponding to Evol concentration of 2.5 nM) were added and cultured for 24 h. BeyoClick™ EdU cell proliferation kit (RiboBio) was used to label EdU positive cells.

#### Migration assay of VSMCs

24-well plates were seeded with VSMCs. After stimulating with LPS (100 ng/mL) for 24 h, Evol, Lipo@E NPs, and (Lipo + M)@E NPs (at the same Evol concentration of 2.5 nM) were added and cultured for 24 h. Then, the upper chamber was seeded with VSMCs (1 × 10^5^ cells/chamber). After 24 h, removing the cells in the upper chamber, staining the migrated cells with 0.1% crystal violet. In addition, wound healing assay was also performed to investigate VSMCs migration. VSMCs were cultured into 12-well plates until the monolayer was fully confluent. The cells were scraped and starved for 6 h. After washing with PBS, serum-free medium containing Evol, Lipo@E NPs, and (Lipo + M)@E NPs were added and incubated for 24 h later. Finally, the images of cell migration were captured under inverted microscope (Olympus, IX-73).

#### Cellular uptake of oxLDL and Oil Red O staining of VSMCs

After stimulating by LPS (100 ng/mL) for 24 h, VSMCs were incubated with Evol, Lipo@E NPs, and (Lipo + M)@E NPs (at the same Evol concentration of 2.5 nM) for another 2 h. Then, 40 μg/mL DiL-oxLDL (free fetal bovine serum) was added and incubated for 4 h. Afterwards, CLSM was used to observe fluorescence images. In addition, after treatment with LPS (500 ng/mL) for 24 h, VSMCs were incubated with different NPs for 2 h, then 80 μg/mL oxLDL was added to the medium. Fresh medium was used as the Control group, and oxLDL was used as the Model group. The cells were stained with 0.3% ORO after 48 h.

#### Immunofluorescence assay

The VSMCs were treated with Evol, Lipo@E NPs, and (Lipo + M)@E NPs (Evol concentration of 2.5 nM) for 24 h, and sequentially fixed and permeabilized. After blocking for 30 min, primary antibodies against PCSK9 (1:100)/OPN (1:200)/Vimentin (1:200) were added for 12 h. After washing with PBST 3 times, fluorescence secondary antibody (1:500) was added for 2 h. Nuclei were stained with DAPI before observing under CLSM (Nikon, Ti-E + A1 MP, Japan).

#### Transcriptomics analysis

LPS-treated VSMCs without/with (Lipo + M)@E NPs treatment for 24 h were set as the Model and (Lipo + M)@E group, respectively. Total RNA was extracted from the Model and (Lipo + M)@E group (*n* = 3), which was performed by Biomarker Technologies (Beijing, China).

### *Pharmacokinetics, targeting and permeating capability*

Eyelid blood samples of C57BL/6 mice were collected from different NPs treatments (5 mg/kg, Ce6) at 0, 1, 2, 3, 4, 6, 8, 12, and 24 h after *i.v.* administration. Serum samples were detected by IVIS spectroscopy system (Lumina XR).

After 10 weeks of HFD, ApoE^−/−^ atherosclerotic mice were injected intravenously with PBS, Ce6, Lipo@Ce6 NPs, and (Lipo + M)@Ce6 NPs (5 mg/kg, Ce6). After 12 h, the aortas and other organs of mice were measured by IVIS spectroscopy (Lumina XR).

After treating ApoE^−/−^ atherosclerotic mice with Lipo@E and (Lipo + M)@E NPs for 8 weeks, anti-PEG (TU398602, 1:500, Abmart) was used for immunofluorescence stain of frozen sections of aortic roots as both Lipo@E and (Lipo + M)@E NPs were modified with DSPE-PEG_2000_-NH_2_ to observe the infiltration of nanomaterials in the atherosclerotic plaque.

### *In vivo anti-atherosclerosis study*

ApoE^−/−^ mice with 6-week-old male were fed a high-fatdiet (HFD, 40% fat, 40% carbohydrate, 20% protein, and 1.25% cholesterol; D12108C) for 4 weeks and received intravenous saline, Evol, Lipo@E NPs and (Lipo + M)@E NPs at a dose of 5 mg/kg Evol (twice weekly) for 8 weeks. The mice were continuously fed a HFD during treatment.

#### Oil red O staining of aortas

Aortas of ApoE^−/−^ mice were collected, opened longitudinally and stained with ORO. The aortic root, aortic arch, and abdominal aorta of the aorta, which are prone to plaque formation in ApoE^−/−^ mice, were also collected and transected frozen sections to stain with ORO.

#### Aorta tissues histological study and blood ELISA analysis

The paraffin sections were incubated with primary antibodies including F4/80 antibody (SC-377009, 1:200; Santa Cruz), α-SMA antibody (55135-AP, 1:100; proteintech), MMP-9 antibody (10375-2-AP, 1:200; proteintech), CD31 antibody (11265-1-AP, 1:4000; proteintech), and PCSK9 antibody (55206-1-AP, 1:200; proteintech) for 2 h and secondary antibodies for 1 h at 25 °C. Meanwhile, the paraffin sections were stained with hematoxylin–eosin (H&E) and Masson’s trichrome (G1346, Solarbio), respectively. Furthermore, serum was collected to detect the levels of PCSK9 by ELISA kit (KE10050, proteintech).

#### Metabolomics analysis and microbiological analysis

Blood samples of ApoE^−/−^ mice in the Control group, Model group, and (Lipo + M)@E group were collected and centrifuged to obtain plasma. Plasma samples and colon contents were snap-frozen in liquid nitrogen for 15 min and stored at − 80 °C before analysis.

Metabolomics analysis and Microbiological analysis were performed by Beijing Novogene Technology Co., LTD. Plasma samples were subjected to positive and negative ion metabolomics analysis using an LC–MS system.

### *Biocompatibility and biosafety evaluation of (Lipo + M)@E NPs*

Cytotoxicity assay: VSMCs, HUVECs, and H9C2 cells were seeded into 96-well plates and incubated with medium containing Lipo NPs, Evol, Lipo@E NPs, and (Lipo + M)@E NPs, respectively.

Hemolysis test: Pure RBCs were obtained from the blood of C57BL/6 mice [16]. The erythrocyte suspension (*V*/V) was mixed with Lipo NPs, Evol, Lipo@E NPs, and (Lipo + M)@E NPs (corresponding to lecithin concentrations of 0.9, 1.8, and 3.6 μg/mL), incubated at 37 ℃ for 4 h, the supernatant was collected after centrifugation. Meanwhile, microscope was used to capture the RBC morphology.

Platelet aggregation assay: Whole blood of C57BL/6 mice was collected and centrifugated to obtain platelet rich plasma [17]. Then, Lipo NPs, Evol, Lipo@E NPs, and (Lipo + M)@E NPs (corresponding to 3.6 μg/mL lecithin) were incubated with plasma at 37 ℃ for 1 h. Detect the absorbance values at 650 nm wavelength.

The blood samples and major organs from ApoE^−/−^ mice were collected for safety evaluation.

### Statistical analysis

Data were analyzed by GraphPad Prism version 8.3.0 using one-way analysis of variance (ANOVA). All data were presented as the mean value ± the standard deviation of independent experiments.

## Results and discussion

### Preparation and characterization of (Lipo + M)@E NPs

**Fig. 2:**
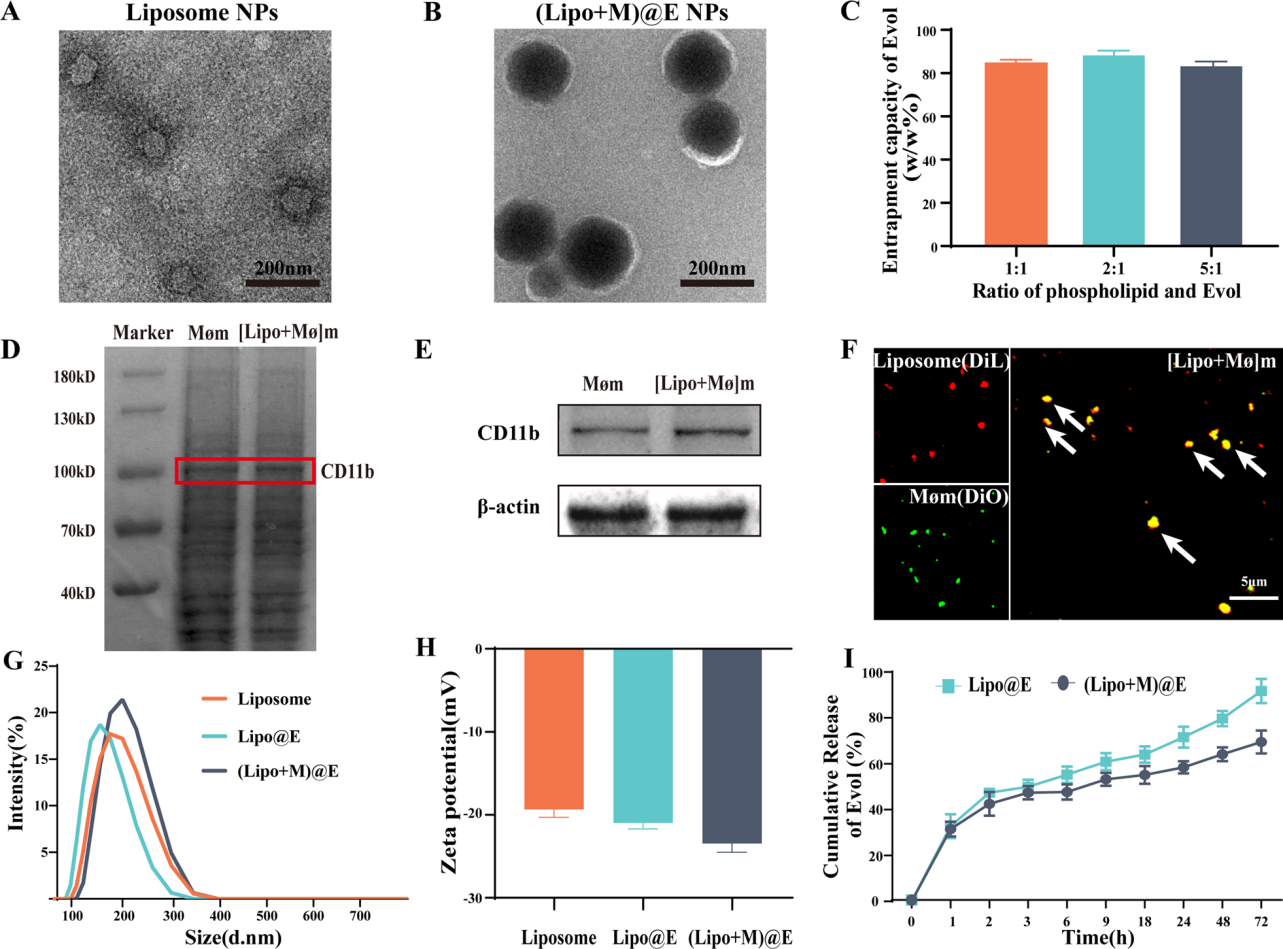
Characterization of (Lipo + M)@E NPs. TEM image of Liposome NPs (**A**) and (Lipo + M)@E NPs (**B**). (**C)** Evol entrapment efficiency of (Lipo + M)@E NPs at the different ratios. (**D)** SDS-PAGE analysis of retention protein bands of Møm and [Lipo + Mø]m NPs. (**E)** Western blot of Møm and [Lipo + Mø]m NPs for characteristic Møm marker CD11b. (**F)** Confocal fluorescence microscopy imaging of a mixture of [Lipo + Mø]m NPs. Red indicated Liposome and green indicated Møm. Scale bar = 5 μm. Particle size (**G**) and zeta potential (**H**) of (Lipo + M)@E NPs were analyzed by DLS. (**I)** Evol release from Lipo@E NPs and (Lipo + M)@E NPs in PBS at pH 7.4

The reverse evaporation method was applied to prepare Liposome NPs and (Lipo + M)@E NPs. TEM images showed spherical morphology of liposomes NPs and (Lipo + M)@E NPs. Meanwhile, a thin film coating around the (Lipo + M)@E NPs appeared after membrane modification (Fig. 2A&B). As shown in Fig. 2C, the encapsulation efficiency (EE %) of (Lipo + M)@E NPs were more than 80% at 1:1, 2:1, and 5:1 of lecithin/Evol. SDS-PAGE assay demonstrated (Lipo + M)@E NPs showed a similar protein profile to Møm (Fig. 2D). By performing western blot, we confirmed that the (Lipo + M)@E NPs expressed the Møm specific marker CD11b (Fig. 2E). In addition, by performing membrane colocalization assay as our previously described method [18], we found that simple mixing of DiO-labeled Møm and DiL-labeled Lipo NPs showed separate green and red fluorescence (Fig. 2F). In contrast, overlapping color (yellow signals) was observed in [Lipo + Mø]m. The above results indicated successful fusion between Møm and Lipo. Additionally, average diameter of (Lipo + M)@E NPs measured by DLS was 183.2 ± 1.6 nm (Fig. 2G) and the zeta potential was -23.46 ± 0.31 mV (Fig. 2H). Finally, we investigated the release behavior of Evol in PBS (pH 7.4) at 37 °C. The release rates of Evol from Lipo@E NPs and (Lipo + M)@E NPs were 85.6% and 64.8% at 72 h, respectively (Fig. 2I). These results demonstrated that cell membrane encapsulating could reduce the release rate of Evol from (Lipo + M)@E NPs, which is beneficial for drug release and disease therapy [19].

### In vitro cellular uptake

**Fig. 3:**
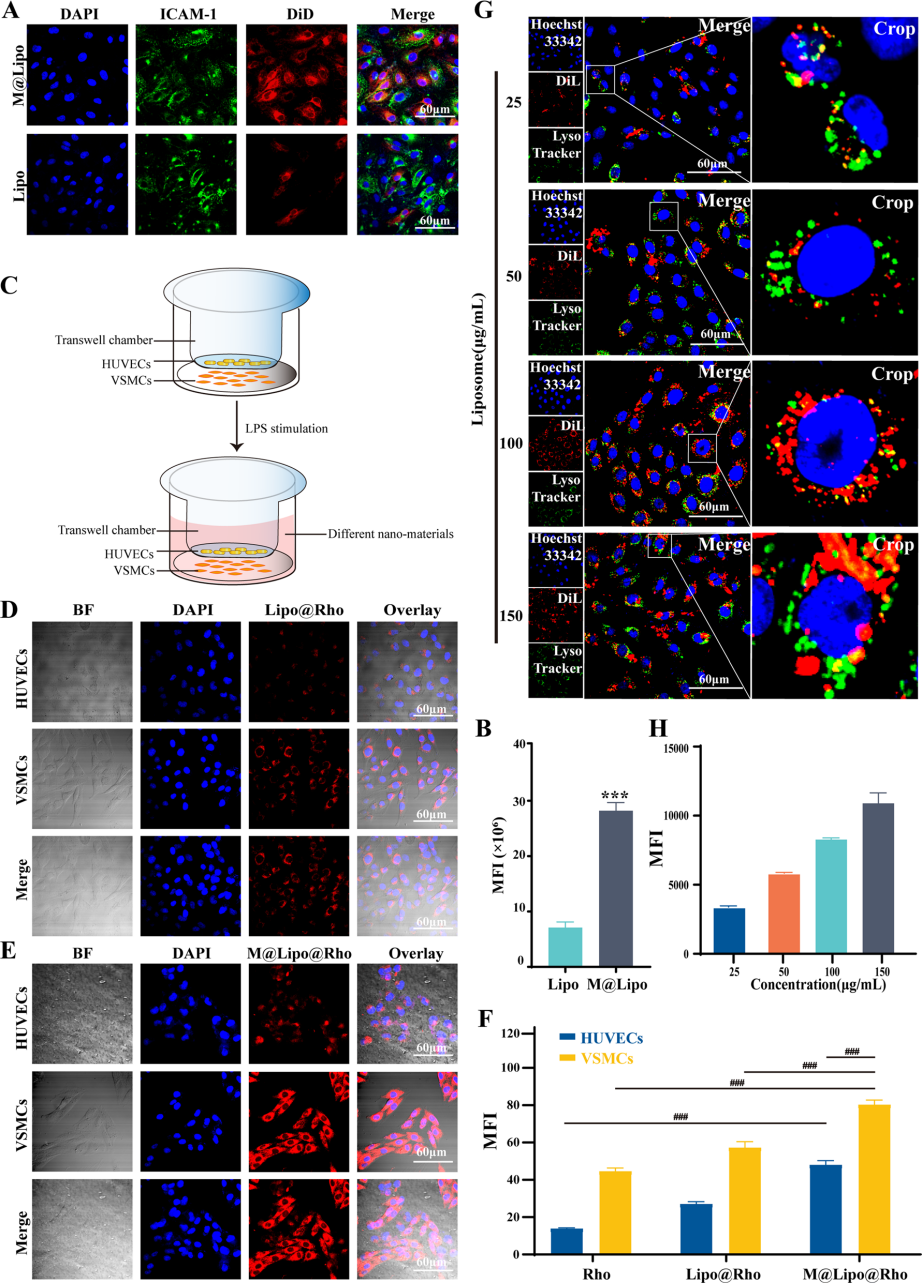
Cellular uptake of M@Lipo NPs. (**A)** Representative photos of Lipo/M@Lipo (red), HUVECs (blue), and ICAM-1 (green) in the LPS-treated group. (**B)** Semi-quantitative analysis of the uptake of different NPs in VSMCs and HUVECs. Data are means ± SD, *n* = 3, ***P < 0.001 vs. Lipo. (**C)** The schematic diagram showed that HUVECs were co-cultured with VSMCs in a transwell system for simulating plaque in vitro. (**D**&**E)** Phagocytosis of Lipo@Rho NPs and M@Lipo@Rho NPs in VSMCs and HUVECs in transwell. BF indicates bright field. (**F)** Fluorescence quantitation of Lipo/M@Lipo (red). Fluorescence images (**G**) and quantitation (**H**) of VSMCs 4 h after incubation with different concentrations of M@Lipo NPs. Scale bars = 60 μm

Under physiological conditions, intercellular cell adhesion molecule-1 (ICAM-1) is lowly expressed in the vascular endothelial cells. However, inflammatory lesion can induce the upregulation of ICAM-1, thus providing chemotactic signals to recruit more macrophages. After binding on the vascular endothelium, macrophages can penetrate the blood duct into the inflammatory plaque [20, 21]. In order to investigate the effect of Møm coating on the interaction between nano-materials and activated endothelial cells, LPS-treated HUVECs were used to simulate the inflammatory microenvironment, which is necessary for the initiation of atherosclerosis. DiD was used to label different nano-materials. The fluorescence signals emitted from DiD indicate the high expression of ICAM-1 in the LPS-treated HUVECs (green). Meanwhile, the recruitment ability of LPS-induced HUVECs to (Lipo + M)@E NPs (red) was significantly higher than that of unmodified nanoparticles (Fig. 3A&B). This result also demonstrated the strong interaction between inflammatory HUVECs with high ICAM-1 expression and Møm coating nano-materials, as CD11b on Møm surface can act as a ligand of ICAM-1 in inflammatory HUVECs [22].

As VSMC-derived cells accounted for more than 70% of atherosclerotic plaque cells, we co-cultured the HUVECs and VSMCs in the LPS-containing transwell chambers to simulate the atherosclerotic plaque environment in vitro. Next, the uptake performance of M@Lipo by VSMCs was investigated to reflect the permeability of M@Lipo in plaque according to the process in Fig. 3C. As shown in Additional file 1: Fig. S1 and Fig. 3D–E, both HUVECs and VSMCs showed stronger phagocytosis to all NPs, comparing with the free Rho group. However, the phagocytosis ability of VSMCs is higher than that of HUVECs for these nano-materials. It should be noted that the phagocytosis ability of VSMCs to M@Lipo NPs was significantly higher than that of Lipo NPs. This result demonstrated that Møm coating could help nanomaterials pass through the HUVECs layer in the small chamber and enter the underlying VSMCs. Therefore, macrophages membrane improved the penetration and targeting ability of (Lipo + M)@E NPs to VSMCs in atherosclerotic plaque (Fig. 3F), which is conducive to increasing the enrichment of Evol at the lesion site.

Lysosomes of VSMCs are organelles that digest outside materials entering into VSMCs. The probe of LysoTracker (green) was used to monitor the location of M@Lipo NPs after entering into VSMCs. The gradually increased fluorescence signal in VSMCs reflected that M@Lipo NPs were taken up by VSMCs in a concentration-dependent manner (Fig. 3G&H), almost no endocytic M@Lipo NPs were transported into VSMCs via the lysosomal pathway, which was confirmed by the weak overlap of red and green (yellow) fluorescence. In addition, the inhibitors assay indicated that the uptake efficacy of VSMCs was reduced by 55% with colchicine (macropinocytosis mediated endocytosis inhibitor) pre-treatment. Therefore, we can speculate that M@Lipo NPs were taken up by VSMCs through the macropinocytosis pathway (Additional file 1: Fig. S2). As macropinocytosis facilitates the lysosomal escape of entrapped materials [23], the internalization of M@Lipo NPs into VSMCs can effectively prevent the drug from lysosomal degradation, thus maintaining its stability.

Macrophages of the reticuloendothelial system are the executor of phagocytosis and clearance of foreign substances in vivo. Avoiding phagocytosis and clearance by macrophages can significantly improve the performance of the drug delivery system [24, 25]. Additional file 1: Fig. S3 showed that the red fluorescence intensity in RAW264.7 cells significantly reduced due to the membrane coating, compared with sole Lipo NPs. These results clearly demonstrated that the membrane coating enables M@Lipo NPs can maximize the retention of drug concentrations by endowing immune escape ability. Moreover, after entering into blood vessels, the encapsulated Møm is conducive to the recruitment of nano-materials by damaged HUVECs to increase drug enrichment in plaque, ultimately achieving improved drug bioavailability to treat atherosclerosis.

### Circulatory time, targeting, and accumulation assay in atherosclerotic lesions

**Fig. 4:**
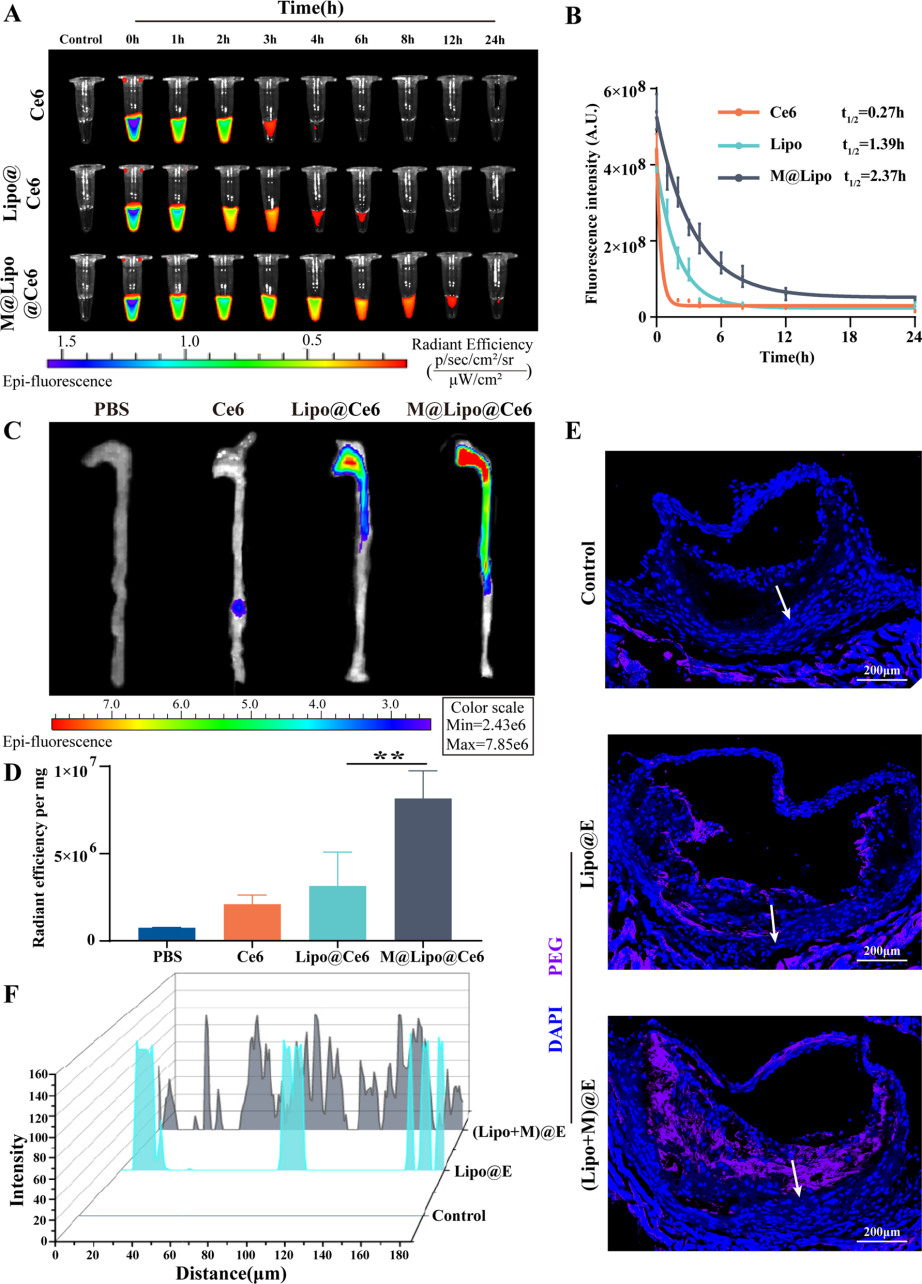
Pharmacokinetics and targeting capability of (Lipo + M)@E NPs. (**A)** Representative photographs of blood samples collected from C57BL/6 mice after administration of different nano-materials at various time points. (**B)** Pharmacokinetic curves of different nano-materials. *n* = 3. Fluorescence photos (**C**) and semi-quantitative (**D**) show the fluorescent signals of Ce6 in aortas from ApoE^−/−^ mice. ApoE^−/−^ mice fed with HFD for 2 months were intravenously injected with different NPs. After administration of 12 h, the aortas of ApoE^−/−^mice were isolated for detection. Data are means ± SD, *n* = 3, ^**^P < 0.01 *vs.* the Lipo@Ce6 NPs. (**E)** Immunofluorescence imaging showing accumulation and infiltration of different nano materials (immunofluorescence stained for anti-PEG) in the aortic roots of ApoE^−/−^ mice after 8 weeks of serial treatment. Blue indicated nuclei and purple indicated nano materials (stained for anti-PEG) in plaque. Scale bars = 200 μm. (**F)** Analysis of (Lipo + M)@E NPs fluorescence intensity (stained for anti-PEG) along the white arrowed lines. *n* = 3

Next, we investigated the effect of macrophages membrane coating on the half-life and accumulation targeting of nanomaterials in vivo. The pharmacokinetics of M@Lipo@Ce6 NPs in C57BL/6 mice were examined via *i.v.* administration. Figure 4A indicated that the fluorescence intensity of blood samples gradually decreased over time during the investigated process (24 h). The quantitative assay demonstrated that the half-time of M@Lipo@Ce6 NPs increased 0.71- and 7.7- fold compared with Lipo@Ce6 NPs (2.37 h *vs.* 1.39 h) and sole Ce6 (2.37 h *vs.* 0.27 h), respectively (Fig. 4B), the consistent result with the previous report [26, 27], demonstrated that Møm coating exhibited superior blood retention. In addition, Fig. 4C indicated that the fluorescence signal enrichment of both nanoparticles at the atherosclerotic plaque, while the signal intensity of M@Lipo@Ce6 NPs treated group increased 1.59-fold, comparing with Lipo@Ce6 NPs (Fig. 4D). However, the accumulation was further improved in Lipo@Ce6 NPs group by utilizing the high affinity between ICAM-1 of injured endothelial cells and CD44 receptor on macrophage membrane [28]. This result demonstrated that passive targeting caused the aggregation of Lipo@Ce6 NPs at the atherosclerotic plaque. In order to assess whether (Lipo + M)@E NPs can improve penetrating ability in atherosclerotic plaque, ApoE^−/−^ atherosclerotic mice were treated according to the protocol in Fig. 7A. At the end of treatment, anti-PEG was used for immunofluorescence staining of frozen aortic roots sections. The result demonstrated that only weak non-specific fluorescent signals in the Control aorta. In the Lipo@E group, most of Lipo@E NPs were found in the plaque margin. Interestingly, after the treatment had finished a week, a significant increase of purple (Lipo + M)@E NPs accumulation was found in the atherosclerotic plaque. Moreover, intense distribution of (Lipo + M)@E was observed in the center of plaque (Fig. 4E). This result clearly demonstrated that Møm-Lipo fusion could enhance the plaque penetration of (Lipo + M)@E. The quantitative fluorescent data indicated significant improvement in the intra-plaque distribution in the (Lipo + M)@E-treated group as compared with the Control and Lipo@E group (Fig. 4F). These results suggest that Møm modification enables long circulation and targeting of M@Lipo NPs in vivo, and enhances penetration and accumulation of M@Lipo NPs in atherosclerotic plaque, which will be able to maintain the release of Evol and increase the concentration of drugs in plaque, thereby slowing the progression of atherosclerosis.

Besides, fluorescence images indicated that M@Lipo@Ce6 NPs mainly enriched in the liver and kidney. Compared with the Lipo@Ce6 group, the M@Lipo@Ce6-treated mice showed a ~ 1.5-fold higher in the liver. Meanwhile, the concentration of M@Lipo@Ce6 in the liver was approximately 12-fold higher compared with other organs in the M@Lipo@Ce6 group (Additional file 1: Fig. S4). Since binding between Evol and LDLR can reduce LDL in the liver [29], this result showed that M@Lipo@Ce6 enrichment in the liver could effectively alleviate atherosclerosis by adjusting lipid metabolism.

### (Lipo + M)@E NPs inhibit VSMCs proliferation and migration by reducing PCSK9

As a strong chemical stimulator, LPS can promote the dedifferentiation and migration of VSMCs [30]. Additional file 1: Fig. S5 showed the upregulation of PCSK9 levels in VSMCs incubated with LPS in a concentration-dependent manner. However, 2.5 nM Evol significantly attenuated PCSK9 levels in 100 ng/mL LPS-induced VSMCs (Additional file 1: Fig. S6), which was considered an appropriate concentration for subsequent studies.

**Fig. 5:**
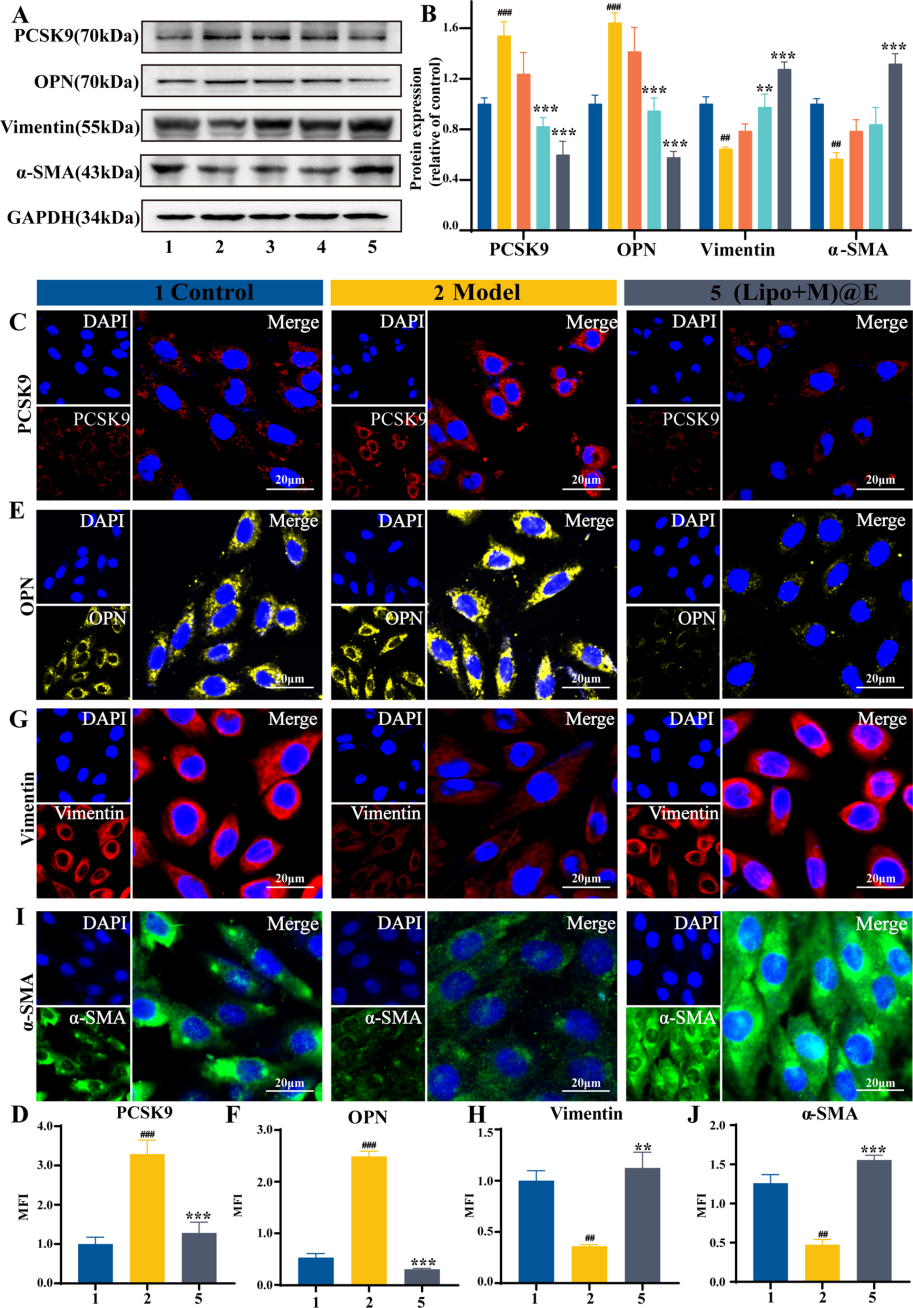
(Lipo + M)@E NPs regulated the phenotypic transformation of VSMCs by reducing PCSK9 levels. (**A)** Represented photograph showing the protein expressions of PCSK9, OPN, Vimentin, and α-SMA in VSMCs after treatment with Evol, Lipo@E NPs, and (Lipo + M)@E NPs. (**B)** The relative quantification analysis of PCSK9, OPN, Vimentin, and α-SMA. Immunofluorescence images and quantitative analysis of PCSK9 (red) (**C**&**D**), OPN (yellow) (**E**&**F**), Vimentin (red) (**G&H**), and α-SMA (green) (**I**&**J**) in VSMCs after treatment with (Lipo + M)@E NPs. Scale bars = 20 μm. 1: Control, 2: Model, 3: Evol, 4: Lipo@E NPs, 5: Lipo + M)@E NPs. Data are means ± SD, *n* = 3, ^##^*P* < 0.01, ^###^*P* < 0.001 *vs.* the Control. ^**^P < 0.01, ^***^P < 0.001 *vs.* the Model

Using western blot assay and immunofluorescence imaging, we detected the change of PCSK9 in VSMCs with (Lipo + M)@E NPs treatment. Figure 5A&B indicated the upregulation of PCSK9 levels in VSMCs with LPS treatment was significantly reversed by the (Lipo + M)@E NPs treatment. Consistent with this result, immunofluorescence images also demonstrated the strong inhibitory effect of (Lipo + M)@E NPs on the PCSK9 expression (Fig. 5C&D).

Since the phenotypic transformation of VSMCs is involved in the progression of atherosclerosis, we then investigated the effect of (Lipo + M)@E NPs on the levels of phenotype markers of VSMCs. Both western blot (Fig. 5A&B) and immunofluorescence analysis (Fig. 5E–J) indicated that (Lipo + M)@E NPs could significantly reverse the levels of a synthetic marker of OPN and contractile markers of α-SMA and Vimentin in VSMCs in vitro. Compared with the Model group, (Lipo + M)@E NPs upregulated the levels of Vimentin and α-SMA in VSMCs, while decreasing the levels of OPN. These results demonstrated that (Lipo + M)@E NPs could regulate the phenotypic switching of VSMCs by down-regulating the expression of PCSK9, thereby alleviating atherosclerosis. Notably, Evol with low dosage inhibited the proliferation and migration of VSMCs, the efficacy is much lower, compared with the (Lipo + M)@E NPs group, as the encapsulation of Møm not only promoted the uptake of nanomaterials by VSMCs but also protected Evol from decomposition and destruction before reaching the destination.

In addition, RNA-sequencing indicated that (Lipo + M)@E NPs treatment could result in the up-regulation of 393 expressed genes and down-regulation of 330 expressed genes, compared with LPS-induced VSMCs (Additional file 1: Fig. S7A). GO functional annotation and analysis indicated that differential expression genes (DEGs) were mainly distributed in the negative regulation of smooth muscle cell proliferation, collagen catabolic process, artery smooth muscle contraction, positive regulation of smooth muscle cell apoptotic process, cell migration, extracellular matrix organization, and response to LPS (Additional file 1: Fig. S7B). In terms of molecular function, they are mainly related to the protein kinase C binding, actin monomer binding, enzyme binding, MAP kinase kinase kinase activity, kinase binding, extracellular matrix structural constituent, collagen binding, and chemokine activity (Additional file 1: Fig. S7C). The cellular components of DEGs were mainly expressed in the actin cytoskeleton, membrane protein complex, endosome, rough endoplasmic reticulum, smooth muscle contractile fiber, receptor complex, membrane raft, extracellular space, and extracellular matrix (Additional file 1: Fig. S7D). GSEA further demonstrated that (Lipo + M)@E NPs down-regulated the levels of genes enriched in actin binding, actin monomer binding, and extracellular matrix structural constituent, while up-regulated the expression of DEGs in negative regulation of cell proliferation (Additional file 1: Fig. S7E). Abnormal migration and proliferation of VSMCs [31], and extracellular matrix (ECM) secretion by the proliferation of VSMCs can cause the pathogenesis of macrovascular diseases. Remodeling of actin cytoskeleton regulates cell motility, migration, and invasion [32, 33]. This remodeling relies on the polymerization of G-actin into F-actin, allowing dynamic regulation of the biomechanical properties of cells [34]. Additional file 1: Fig. S7E showed that (Lipo + M)@E NPs could inhibit the expression of genes in actin and actin monomer binding process. It is known that the contractile type of VSMCs may transform into the synthetic type of VSMCs to improve proliferation, migration, and plaque formation [35]. The above results indicated that (Lipo + M)@E NPs could negatively regulate the proliferation of VSMCs by acting on actin and ECM of VSMCs.

**Fig. 6:**
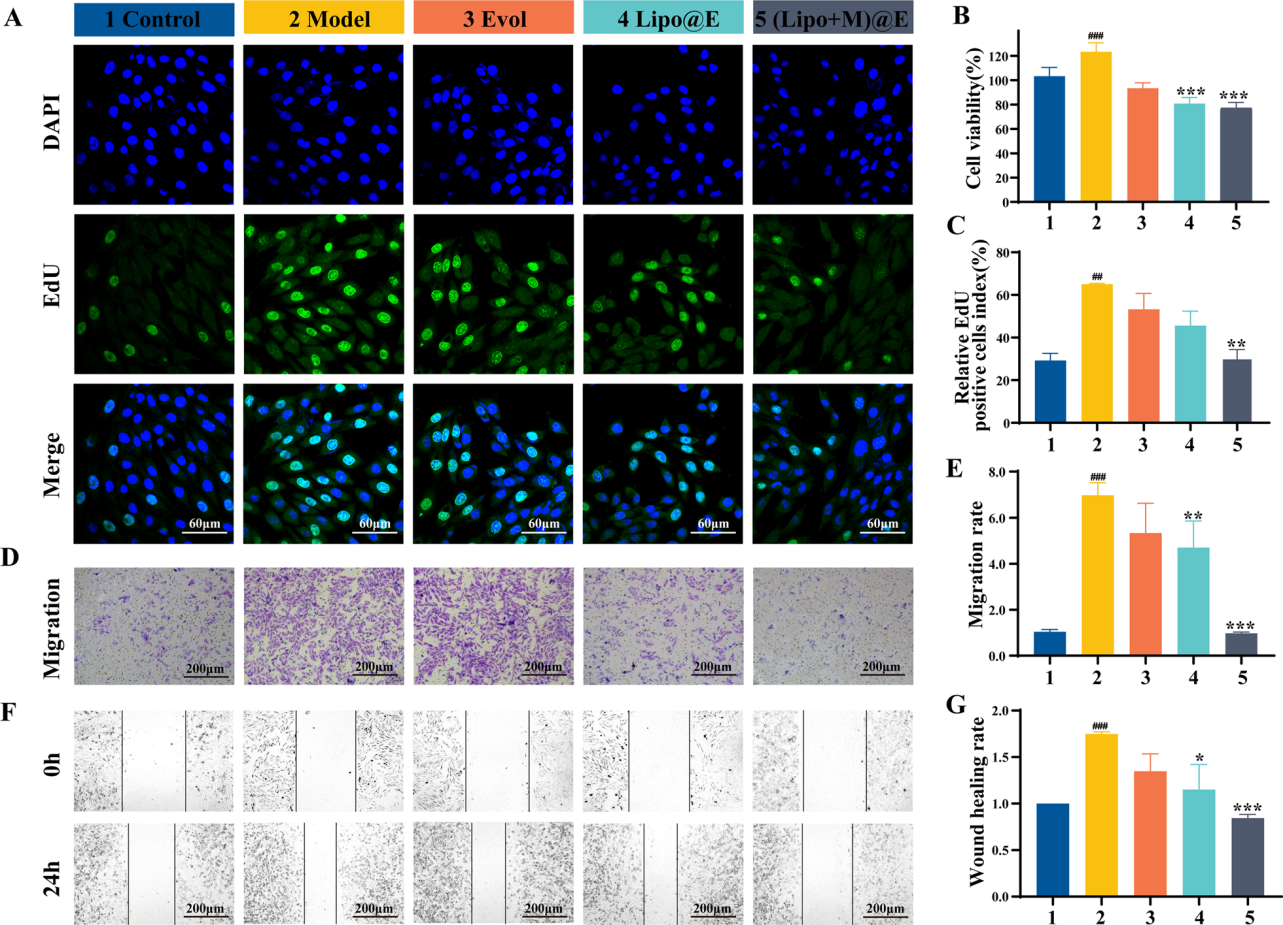
(Lipo + M)@E NPs inhibit VSMCs proliferation and migration. (**A)** The proliferative VSMCs were determined by EdU incorporation assay. Blue represents nuclei and green represents the EdU-positive VSMCs. Scale bars = 60 µm. (**B)** Viability assay of VSMCs with different treatments. (C) The ratio of EdU-positive cells to total cells under different treatments. (**D)** Migrated VSMCs were detected by transwell assay. Scale bars = 200 µm. (E)The number of migrated VSMCs. Images (**F**) and quantitative (**G**) of wound-healing assay of VSMCs with different treatments. Scale bars = 200 µm. Data are means ± SD, *n* = 3, ^##^*P* < 0.01, ^###^*P* < 0.001 *vs.* the Control. ^*^P < 0.05, ^**^P < 0.01, ^***^P < 0.001 *vs.* the Model

Subsequently, we performed viability, proliferation, and migration assay of VSMCs with different treatments. Compared with the Control group, the viability of VSMCs with LPS treatment increased by 19.95%, while the viability of VSMCs decreased by 46.2% in the presence of (Lipo + M)@E NPs, compare with the Model group (Fig. 6B). EdU staining also showed that proportion of EdU-positive cells increased 36% after treatment with LPS, compared with the Control group. In contrast, the proportion of EdU positive cells in VSMCs treated with (Lipo + M)@E NPs decreased by 35%, compared with the Model group (Fig. 6A&C). In addition, Fig. 6D&E indicated that LPS strongly promoted VSMCs migration, with a migration rate 7.14-fold higher than the basic levels, while (Lipo + M)@E NPs reduced migration to 15.7%. Wound healing assay also indicated the promotion of LPS on the migration of VSMCs (increased by 42.6%), compared with the Control group. On the contrary, the migration rate of the (Lipo + M)@E NPs group decreased by 51.1%, compared with the Model group (Fig. 6F&G). These results suggested that (Lipo + M)@E NPs could significantly inhibit the proliferation and migration of VSMCs induced by LPS.

As 70% of foam cells in coronary atherosclerosis are mainly derived from VSMCs [36, 37]. We then investigate the effect of (Lipo + M)@E NPs on the uptake behavior of VSMCs to DiL-oxLDL. Additional file 1: Fig. S8A&B showed stronger red fluorescence in the LPS-induced VSMCs with DiL-oxLDL incubation (increased by 11.8-fold), compared with the Control group. On the contrary, only weak red fluorescence signal appeared because of the effective inhibition of (Lipo + M)@E NPs on the uptake of DiL-oxLDL(decreased by 90%, compared with the Model group). Oil-red O staining similarly demonstrated that LPS significantly promoted lipid droplets accumulation in VSMCs (increased by 16.8-fold, compared with the Control group), while (Lipo + M)@E NPs alleviated the lipid droplets accumulation by about 79%, compared with the Model group (Additional file 1: Fig. S8C&D). The above results suggested that (Lipo + M)@E NPs efficiently inhibited VSMCs-derived foam cells formation by reducing the uptake and internalization of oxLDL.

### Therapeutic efficacy of (Lipo + M)@E NPs against atherosclerosis

**Fig. 7:**
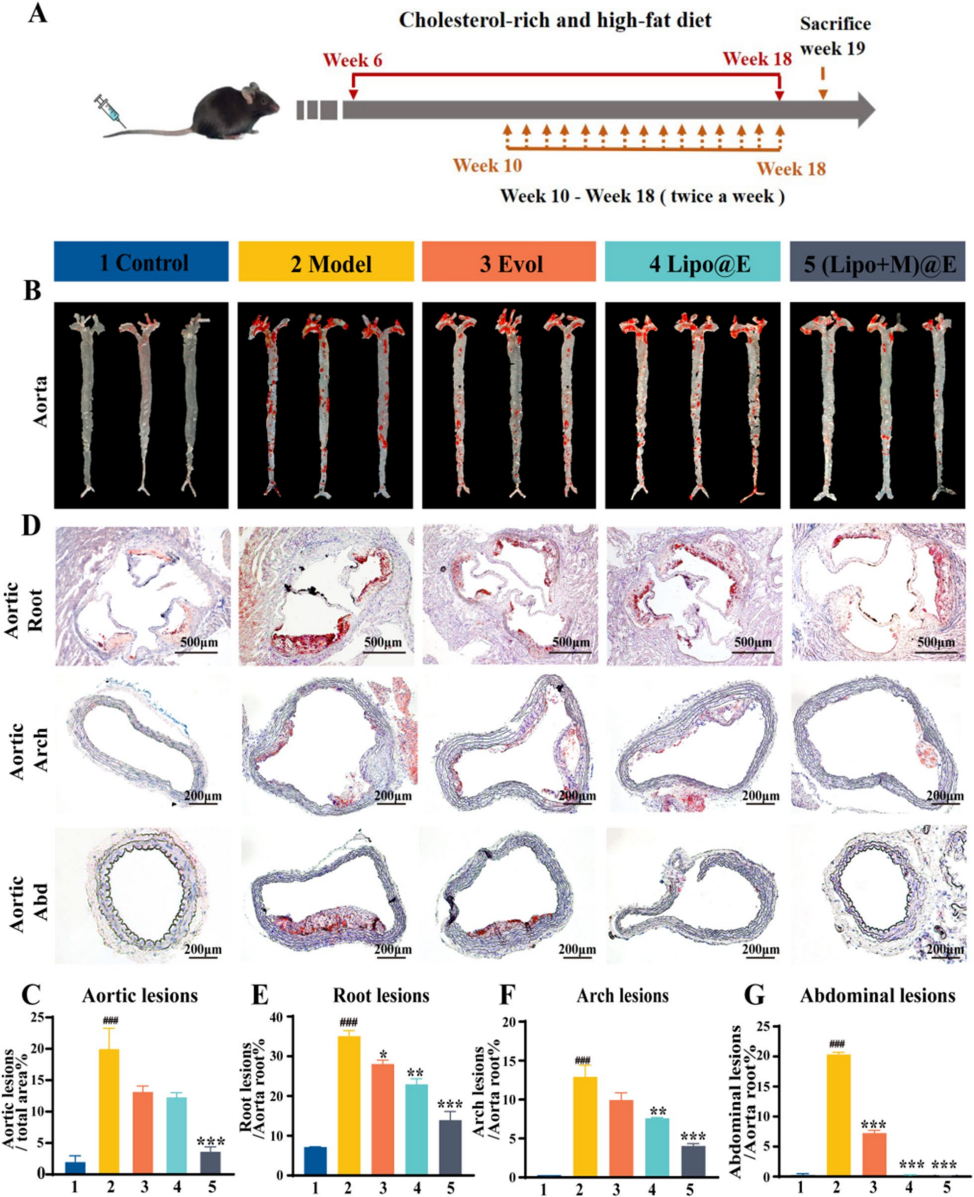
Treatment with (Lipo + M)@E NPs significantly alleviates the progression of atherosclerosis. (**A)** Treatment protocols of ApoE^−/−^ mice. ApoE^−/−^ mice were fed an HFD for 3 months. From the second month, all mice received different treatments by *i.v.* injection twice weekly for the rest of the period. Mice in the Control group were treated with saline alone, while other groups were separately administered with Evol, Lipo@E NPs, and (Lipo + M)@E NPs at the same Evol concentration (5 mg/kg). Representative photograph (**B**) and quantitative (**C**) of en face ORO-stained aortas. (**D**) ORO-stained frozen sections of the aortic root, arch, and abdominal aorta. Quantitative of the relative plaque area in sections of the aorta root (**E**), aortic arch (**F**), and abdominal aorta (**G**). ApoE^−/−^ mice were fed an HFD for 3 months. From the second month, all mice received different treatments by *i.v.* injection twice weekly for the rest of the period. Mice in the Control group were treated with saline alone, while other groups were separately administered with Evol, Lipo@E NPs, and (Lipo + M)@E NPs at the same Evol concentration (5 mg/kg). Scale bars = 500 µm. 1: Control, 2: Model, 3: Evol, 4: Lipo@E NPs, 5:(Lipo + M)@E NPs. Data are means ± SD, *n* = 3, ^###^*P* < 0.001 *vs.* the Control. ^*^P < 0.05, ^**^P < 0.01, ^***^P < 0.001 *vs*. the Model

Based on the above results, in vivo study was performed to evaluate the therapeutic efficacy of (Lipo + M)@E NPs on ApoE^−/−^ atherosclerosis mice according to the protocol of Fig. 7A. ORO staining of aorta revealed that the area of lipid deposited plaque was about 2.08% and 20.14% in the Control and Model group, respectively, the data of which confirmed the successful construction of the atherosclerosis Model. However, the plaque area was differentially reduced in the ApoE^−/−^ mice with treatments, compared with the Model group. Compared with the free Evol group (~ 13.33%) and Lipo@E NPs group (~ 12.44%), (Lipo + M)@E NPs showed the strongest inhibitory effect on aortic plaque formation with plaque area of ~ 3.78% (Fig.7B&C), which was consistent with previous reports [38]. This result demonstrated that this biomimetic nanoparticle could actively target, accumulate and penetrate into plaque. In addition, we studied the effect of (Lipo + M)@E NPs on the plaque formation of aorta, the high occurrence region. ORO staining of the frozen section indicated significant lipid deposition in the plaque of the Model group, while the (Lipo + M)@E NPs showed the most significant anti-lipid deposition effect in all segments of aorta (Fig. 7D–G). Collectively, these results demonstrated the significant therapeutic effect of (Lipo + M)@E NPs on atherosclerosis.

**Fig. 8:**
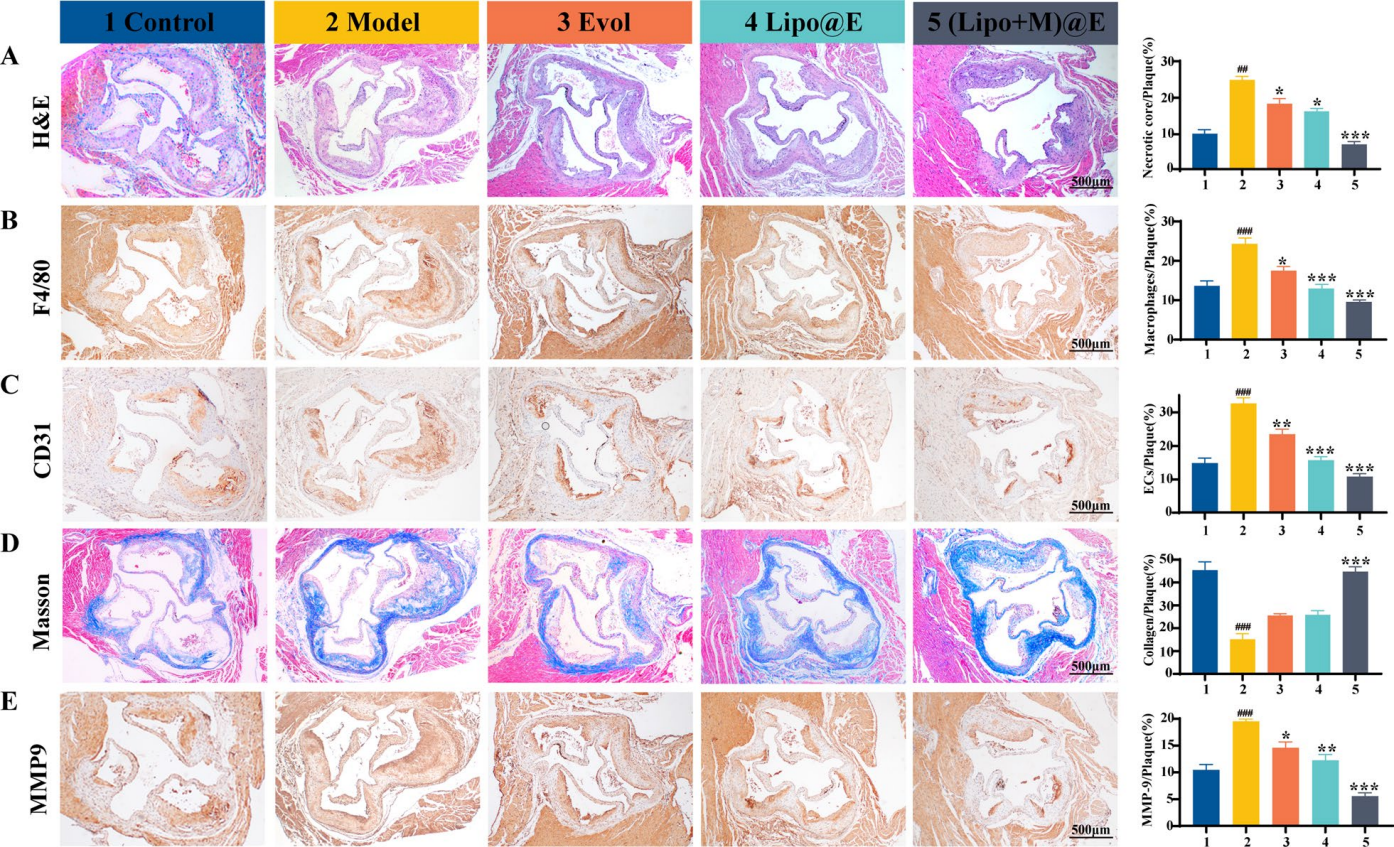
Histochemistry analyses of aortic root paraffin sections from ApoE^−/−^ mice with different treatments. Representative photographs and quantitative analysis of aortic root sections stained by H&E (**A**), antibody to F4/80 (**B**), antibody to CD31 (**C**), Masson’s trichrome (**D**), and MMP9 (**E**). 1: Control, 2: Model, 3: Evol, 4: Lipo@E NPs, 5:(Lipo + M)@E NPs. Scale bars = 500 µm. Data are means ± SD, *n* = 3, ^##^*P* < 0.01, ^###^*P* < 0.001 *vs*. the Control. ^*^P < 0.05, ^**^P < 0.01, ^***^P < 0.001 *vs*. the Model

The proportion of the necrotic core directly determines the vulnerability of plaque [39]. H&E staining indicated that the aortic root plaque with vulnerability was composed of necrotic cores with abundance of lipids in the Model group, and the proportion of necrotic core (24.35%) was higher than that of the Control group (9.65%). However, the proportion of necrotic core was differently reduced in all treatment groups. Specifically, the proportion in the mice with (Lipo + M)@E NPs treatment was only 6.7% (Fig. 8A). In view of the close relationship between the necrotic core and macrophages infiltration in the plaque [40], the immunochemistry method was used to detect the effect of (Lipo + M)@E NPs treatment on the macrophages infiltration. As we predicted, the number of macrophages (represented by F4/80) decreased by 60.8% with (Lipo + M)@E group, compared with the Model group (Fig. 8B). In addition, we detected the CD31 levels of surrounded blood vessels as arterial endothelial dysfunction with high CD31 levels is another important factor in the progression of atherosclerosis [41]. Figure 8C indicated that the levels of CD31 + endothelial cells around blood vessels decreased by 67% after (Lipo + M)@E NPs treatment. Next, we evaluated the effect of (Lipo + M)@E NPs treatment on plaque stability. Masson's trichrome staining showed that (Lipo + M)@E NPs increased the collagen content around the plaque for 1.94-fold, while inhibiting the expression of MMP-9, compared with the Model group (Fig. 8D&E). Stable plaque lesions generally have a thick collagen-rich fibrous cap covering a plaque core, and MMP-9 may promote the development of vulnerable lesions [42, 43]. These results indicated that (Lipo + M)@E NPs significantly reduced plaque area and notably enhanced the stability of atherosclerotic lesions simultaneously, after two months of treatment in ApoE^−/−^ mice with established atherosclerosis.

**Fig. 9:**
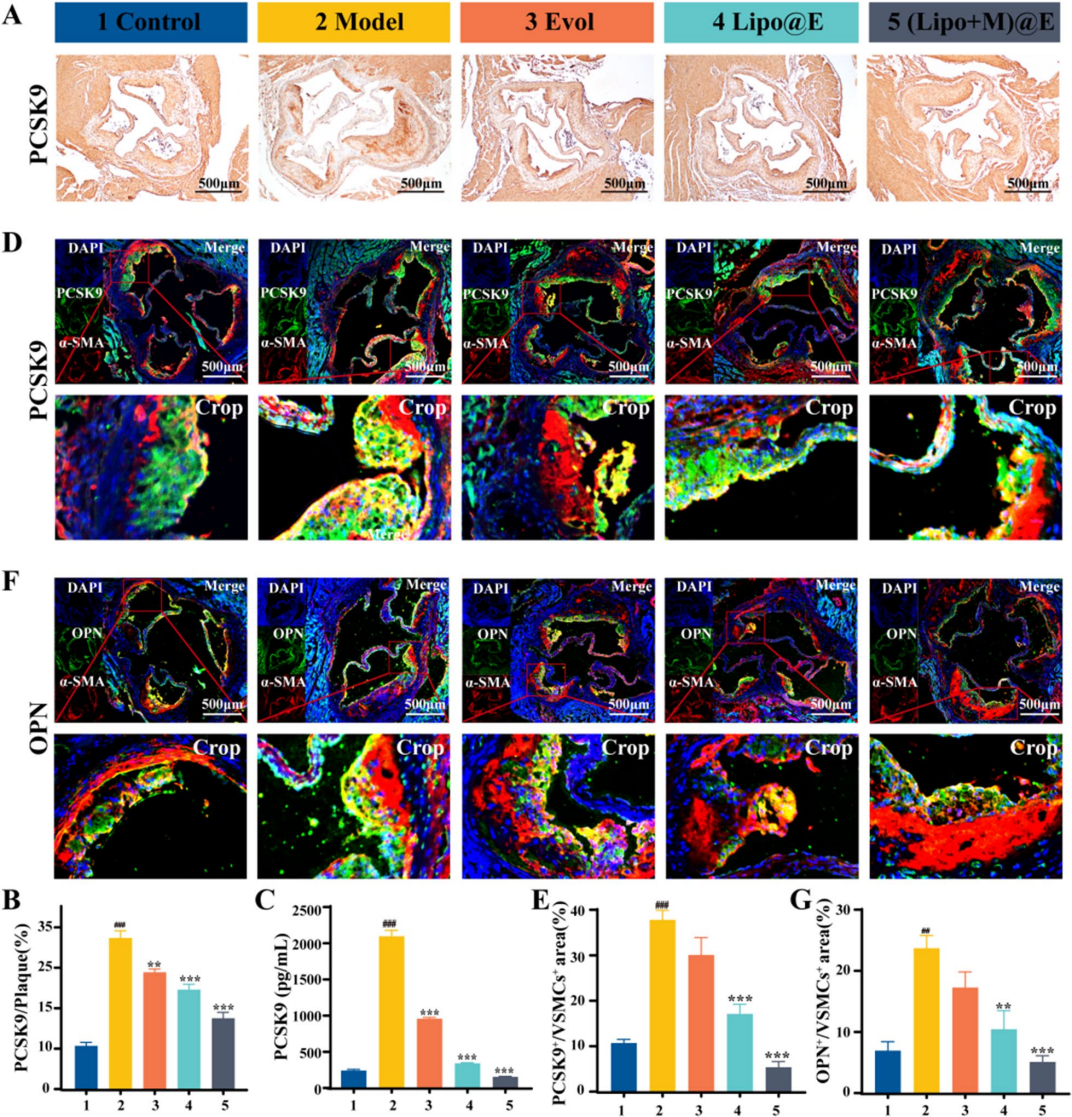
The effect of (Lipo + M)@E NPs on the levels of PCSK9 and OPN in ApoE^−/−^ atherosclerotic mice. Histochemistry photographs (**A**) and quantitative (**B**) of aortic root paraffin sections stained with anti-PCSK9. (**C)** Serum PCSK9 levels in ApoE^−/−^ mics after different treatments were detected by Elisa. (**D)** Frozen sections of aortic root were prepared for double immunofluorescence staining. Green indicated PCSK9, red indicated α-SMA, and yellow indicated PCSK9-positive VSMCs in plaque. (**F)** Immunofluorescence staining using anti-OPN (green) and anti-α-SMA (red), yellow indicated OPN-positive VSMCs in plaque. (**E**&**G)** Quantification analysis of PCSK9 and OPN in VSMCs. 1: Control, 2: Model, 3: Evol, 4: Lipo@E NPs, 5:(Lipo + M)@E NPs. Scale bars = 500 µm. Data are means ± SD, *n* = 3, ^##^*P* < 0.01, ^###^*P* < 0.001 *vs*. the Control. ^*^P < 0.05, ^**^P < 0.01, ^***^P < 0.001 *vs*. the Model

Based on the favorable effect of (Lipo + M)@E NPs on atherosclerosis, we investigated whether (Lipo + M)@E NPs can inhibit PCSK9 levels and phenotypic transition of VSMCs in atherosclerotic mice. Firstly, we examined the intraplaque PCSK9 levels by immunohistochemistry and found that PCSK9 levels were significantly higher in the Model group, compared with the Control group, while reversed by (Lipo + M)@E NPs treatment (37.4% *vs.* 17.5%) (Fig. 9A&B). In addition, by comparing the levels of PCSK9 in the blood of ApoE^−/−^mice with different treatments, we found that (Lipo + M)@E NPs effectively reduced the levels of PCSK9 of Model mice from 2096.19 pg/mL to 156.21 pg/mL, the concentration of which is close to the Control group (242.27 pg/mL) (Fig. 9C). These results definitely demonstrated that (Lipo + M)@E NPs can delay atherosclerosis development by downregulating PCSK9 in VSMCs and in blood. Co-staining of α-SMA and PCSK9 in the VSMCs of aortas plaque also demonstrated a significant ratio increase of PCSK9 positive VSMCs in the Model group, compared with the Control group (37.80% *vs.* 10.70%) (Fig. 9D&E), the phenomenon can be reversed by the (Lipo + M)@E group (the ratio of 5.37%). Next, we investigated the effect of PCSK9 down-regulation on OPN, a synthetic phenotypic marker in VSMCs. Figure 9F&G showed that high expression of OPN in VSMCs in aortic roots of HFD mice, which was revealed by the distinct yellow fluorescence (23.7%). However, the downregulation of PCSK9 caused by (Lipo + M)@E NPs directly result in the decrease of OPN levels by about 71%, which was revealed by the weak yellow fluorescence. These results suggest that cardiac aortic VSMCs phenotypic transformation during the development of atherosclerosis can be reversed by (Lipo + M)@E NPs through inhibiting PCSK9 expression. As the inhibitory effect of Evol on PCSK9 can upregulate LDLR in the liver [29], we then explored the effect of (Lipo + M)@E NPs accumulation in the liver on the LDLR. Western blot showed that intravenous injection (Lipo + M)@E NPs resulted in approximately doubling the LDLR levels in the liver by inhibiting the expression of PCSK9, compared with Evol or Lipo@E NPs (Additional file 1: Fig. S9). This is presumably due to the higher enrichment of M@Lipo NPs in the liver compared with Lipo NPs, which is helpful for alleviating atherosclerosis.

### Effects of (Lipo + M)@E NPs on the intestinal microbiota of ApoE^−/−^ mice

Many studies demonstrated the important role of gut flora in atherosclerosis [44]. Some researchers even took an imbalance of intestinal flora as an independent risk factor for atherosclerosis [45]. Therefore, we used 16S rDNA sequencing to observe the changes of intestinal microbiota in ApoE^−/−^ mice after treatment with (Lipo + M)@E NPs. Simpson and Shannon index showed the good diversity and evenness of species distribution among the communities of the three groups (Additional file 1: Fig. S10A&B). Principal coordinate analysis (PCoA) evaluation showed that the samples of the Model group were completely separated from the Control group, while the samples of the (Lipo + M)@E group were similar to the Control group (Additional file 1: Fig. S10C). These results indicated that the significant change in the overall structure and composition of intestinal microbiota in atherosclerotic mice can be reversed by (Lipo + M)@E NPs. In addition, we also found that intestinal Bacteroidetes and Firmicutes were the dominant bacteria in three groups at the phylum levels, and the ratio of Firmicutes to Bacteroidetes was higher in the Model group, compared to the Control group (Additional file 1: Fig. S10D&E). The (Lipo + M)@E group reversed this tendency by upregulating the abundance of Bacteroidetes and downregulating the abundance of Firmicutes (Additional file 1: Fig. S10F&G). It was reported that the ratio increasing of Firmicutes to Bacteroides and the decrease of Bacteroides number in patients with atherosclerosis [46]. Our research suggested that (Lipo + M)@E NPs could exhibit a benefit modulation of the microbial composition at the phylum levels. At the genus levels, the abundance of Bacteroides in the atherosclerosis group decreased significantly. On the contrary, (Lipo + M)@E NPs treatment could significantly upregulate the abundance of Bacteroides (Additional file 1: Fig. S10H&I). As an important flora to maintain intestinal microbial homeostasis, changes in Bacteroidetes may lead to atherosclerosis [47, 48]. In this study, the abundance of Muribaculaceae was significantly increased in the (Lipo + M)@E group at the genus levels. It was reported that the high abundance of Muribaculaceae in healthy humans or mice can be reduced by a high fat diet [49], while increasing their abundance can lower cholesterol levels and improve microbiota disorders [50]. These results suggested that the therapeutic function of (Lipo + M)@E NPs against atherosclerosis was at least partly through increasing the abundance of Muribaculaceae. These results suggested that (Lipo + M)@E NPs could improve atherosclerosis by regulating lipid metabolism and restoring intestinal homeostasis.

### Effects of (Lipo + M)@E NPs on the levels of serum metabolites in ApoE^−/−^ mice

In addition, metabolomics was performed to investigate the effect of (Lipo + M)@E NPs on the metabolism of ApoE^−/−^ atherosclerotic mice. The negative ion mode of volcano map demonstrated the up-regulation of 22 metabolites and down-regulation of 29 metabolites in atherosclerotic mice compared with Control mice. However, up-regulation of 30 metabolites and down-regulation of 26 metabolites appeared in the (Lipo + M)@E NPs treated mice compared with Model mice. In positive ion mode, 13 metabolites were up-regulated and 20 metabolites down-regulated in atherosclerotic mice relative to Control mice, while 15 metabolites were up-regulated and 30 metabolites down-regulated in (Lipo + M)@E NPs treated mice relative to Model mice (Additional file 1: Fig. S11A). The PLS-DA diagram showed that the well separation among the Control, Model, and (Lipo + M)@E NPs group resulted in the achievement of good clustering (Additional file 1: Fig. S11B). This indicates that metabolomics can be reliably applied to atherosclerosis diagnosis [51]. Additional file 1: Fig. S11C demonstrated the significant difference of metabolites between the Control *vs.* Model group and Model *vs.* (Lipo + M)@E group in the two ion modes. In addition, the Venn diagrams in the two modes were separately drawn to compare the common and unique differences of these groups. In negative ion mode, 51 different metabolites were found between the Control and Model group, while 56 different metabolites were found in the Model and (Lipo + M)@E group. Among them, 25 common differential metabolites were found between the three groups. In positive ion mode, there were 33 differential metabolites between the Control and Model groups, 45 differential metabolites between the Model and (Lipo + M)@E group, and 21 common differential metabolites between the three groups (Additional file 1: Fig. S11D). Finally, by enriching the co-existed metabolites of three groups, we found that the common metabolic pathways of the three groups are mainly Bile acid secretion, Primary bile acid biosynthesis, and Cholesterol metabolism (Additional file 1: Fig. S12A). In addition, Additional file 1: Fig. S12B–D indicated that the levels of Taurochenodeoxycholic acid, Chenodeoxycholic acid, and Deoxycholic acid, which were tightly related to the synthesis of Primary bile acids, decreased significantly in the Model group. However, the PCSK9 downregulation caused by (Lipo + M)@E NPs in turn significantly increased their levels. In addition, Ursodeoxycholic acid was reported to alleviate atherosclerosis in mice by regulating cholesterol metabolism [52], while Palmitoylethanolamide can reduce vascular injury and inflammation in atherosclerosis [53]. Moreover, by detecting the serum total cholesterol (TC) and triglyceride (TG) contents of ApoE^−/−^ mice fed with HFD, we found that the TC and TG contents of (Lipo + M)@E mice were significantly reduced, compared with the Model group (Additional file 1: Fig. S13). The decrease of the metabolites in bile acid synthesis and cholesterol metabolites in the Model group was reversed by inhibiting PCSK9 (Additional file 1: Fig. S12E&F), which means that the inhibition of (Lipo + M)@E NPs on PCSK9 promoted the excretion of bile acids, thereby significantly increasing cholesterol consumption and reducing the risk of atherosclerosis. Apart from this, Pantothenic acid, a common metabolite of Vitamin metabolism, Pantothenate and CoA biosynthesis, and Beta-Alanine metabolism, significantly reduced in the Model group (Additional file 1: Fig. S12G).

In summary, (Lipo + M)@E showed an excellent anti-atherosclerosis effect in ApoE^−/−^mice fed an HFD by regulating the gut microbiota and bile acid metabolism. Our study showed that (Lipo + M)@E NPs could not only inhibit PCSK9 from competitively binding LDLR to LDL, so that more LDLRs could transport LDL to lysosomes for degradation but also unblock the metabolic pathway of converting cholesterol into bile acids in the liver, reduce cholesterol deposition in the liver and promote cholesterol metabolism by regulating the abundance of intestinal flora. The comprehensive regulation made circulating lipids easier to enter the liver for metabolism (Additional file 1: Fig. S14).

### Biocompatibility and biosafety evaluation of (Lipo + M)@E NPs

We investigated the biocompatibility of (Lipo + M)@E NPs by different methods. Firstly, the hemolysis test showed that the hemolysis rate of (Lipo + M)@E NPs was only 3.5% even at the concentration of 20 μM (Additional file 1: Fig. S15A). In addition, no morphological change was observed in erythrocytes with (Lipo + M)@E NPs treatment (Additional file 1: Fig. S15B). Meanwhile, little risk of platelet aggregation in Evol, Lipo@E NPs, and (Lipo + M)@E NPs group (Additional file 1: Fig. S15C). MTT also indicated that after incubating (Lipo + M)@E NPs with VSMCs, HUVECs, and H9C2 cells for 24 h, cell viabilities were above 80%, indicating low toxicity of Lipo + M)@E NPs (Additional file 1: Fig. S15D). These results indicated that (Lipo + M)@E NPs have high biosafety in vivo because of the macrophages membrane coating. In addition, H&E staining indicated that the hepatic lobules of mice in the Control group had clear structures and liver cells were arranged neatly. The liver cells of ApoE^−/−^ mice fed HFD showed round vacuoles of different sizes. However, liver steatosis was significantly improved after (Lipo + M)@E NPs treatment. At the same time, there was no obvious tissue degeneration or obvious injury in other organs including the heart, spleen, lung, and kidney (Additional file 1: Fig. S16A), indicating no injury or obvious side effects during treatment. Blood routine indexes and Liver-kidney assays were within the normal range in (Lipo + M)@E NPs treated mice (Additional file 1: Fig. S16B&C). The biosafety of (Lipo + M)@E NPs was confirmed by the serum levels of liver and kidney function markers in mice. These results indicated the biosafety of (Lipo + M)@E NPs.

## Conclusion

In the current study, we described the development of a (Lipo + M)@E nanoparticle to targeted deliver Evol into VSMCs of atherosclerotic plaque. The key findings of our study are that this compound (1) has good bionic properties, escapes immune cells phagocytose, and has long circulation time, (2) has good targeting properties, penetrates, and accumulates in atherosclerotic plaque. In addition, by inhibiting the proliferation and migration ability of VSMCs, the consequence of phenotypic conversion inhibition of VSMCs, this nano-drug showed the strong therapeutic effect on atherosclerosis treatment, significantly reducing plaque and circulating PCSK9 levels after 8 weeks of treatment. More importantly, this study demonstrated that (Lipo + M)@E NPs can delay the progression of atherosclerosis by regulating gut microbiota and bile acid metabolism, thereby regulating cholesterol metabolism. In our point, this study, which expanded the mechanism of PCSK9 inhibition on atherosclerosis therapy, demonstrated that the constructed (Lipo + M)@E NPs provide a promising option for the treatment of atherosclerosis.

## Supplementary Information

**Additional file 1.** **Fig. S1** Phagocytosis of Rho in VSMCs and HUVECs in a transwell. BF indicates bright field. **Fig. S2** Cell uptake mechanism of M@Lipo NPs. CLSM image (A) and quantitation (B) of the VSMCs uptake for M@Lipo NPs in different inhibitor groups. Scale bar = 60 μm. Data are means ± SD, *n* = 3, ^*^P < 0.05, ^**^P < 0.01, ^***^P < 0.001 *vs.* the Control. **Fig. S3** Immune-escape properties of (Lipo+M)@E NPs in vitro. Confocal images (A) and mean fluorescence intensity (MFI) (B) of different concentrations of Lipo NPs and M@Lipo NPs phagocytosed by RAW264.7 cells. Scale bars = 60 μm. Data are means ± SD, *n* = 3, *P < 0.05, **P < 0.01, ***P < 0.001 *vs*. the Lipo. **Fig. S4** Distribution of M@Lipo NPs in major organs of ApoE^-/-^ mice. (A) Fluorescence imaging of the major organs of ApoE^-/-^ mice with different treatments for 12 h. (B) The relative fluorescence signal of major organs (*n* = 3). Statistically significant differences between M@Lipo@Ce6 NPs in different organs and in the livers (^###^*P* < 0.001); statistically significant differences between Lipo@Ce6 NPs and M@Lipo@Ce6 NPs in the livers (^*^P < 0.05). **Fig. S5** LPS can increase the expression of PCSK9 in VSMCs. LPS-induced expression of PCSK9 in VSMCs in a dose-dependent fashion (measured by western blot). Data are means ± SD, *n* = 3, ^#^*P* < 0.05, ^##^*P* < 0.01 *vs.* the Control. **Fig. S6** Evol can reduce the expression of PCSK9 in VSMCs. Western blot assay of the levels of PCSK9 in VSMCs treated with different concentrations of Evol (2.5 nM, 5.0 nM, and 10.0 nM). Data are means ± SD, *n* = 3, ^###^*P* < 0.001 *vs.* the Control. ^**^P < 0.01, ^***^P < 0.001 *vs.* the Model. **Fig. S7** Transcriptomic analysis of the Model and (Lipo+M)@E group. (A) Volcano plots show differential expression genes between the Model and (Lipo+M)@E NPs group. Red and blue represent genes upregulation and downregulation, respectively. Biological process (B), Molecular function (C), and Cellular component (D) in GO function of the Model and (Lipo+M)@E NPs group. (E) GSEA enrichment plots of differentially expressed genes in the Model and (Lipo+M)@E group. (a) actin binding. (b) actin monomer binding. (c) extracellular matrix structural constituent. (d) negative regulation of cell proliferation. *P* < 0.05. **Fig. S8** Cellular uptake of oxLDL and Oil Red O staining of VSMCs. Representative fluorescence images (A) and quantification (B) of DiL-oxLDL uptake in VSMCs after incubation for 4 h. Scale bars = 60 µm. Images (C) and quantification (D) of oxLDL internalization in VSMCs. Scale bars = 20 µm. Data are means ± SD, *n* = 3, ^###^*P* < 0.001 *vs.* the Control. ^***^P < 0.001 *vs.* the Model. **Fig. S9**. (Lipo+M)@E NPs regulated LDLR and PCSK9 levels in livers of ApoE^-/-^ mice. (A) Represented photograph showing the protein expressions of LDLR and PCSK9 in livers of ApoE^-/-^ mice after treatment with Evol, Lipo@E NPs, and (Lipo+M)@E NPs. (B) The relative quantification analysis of LDLR and PCSK9. Data are means ± SD, *n* = 3, ^###^*P* < 0.001 *vs.* the Control. ^***^P < 0.001 *vs.* the Model. **Fig. S10** (Lipo+M)@E NPs can alter the composition of intestinal flora in ApoE^-/-^ atherosclerosis mice. (A&B) Shannon indexes and Simpson indexes of the Control, Model, and (Lipo+M)@E NPs group. (C) PCoA analysis of the Control, Model, and (Lipo+M)@E NPs group. The relative abundance of gut microbiota at phylum levels (D) and genus levels (H) in the Control, Model, and (Lipo+M)@E NPs groups. (E) The F to B ratio of three groups at the phylum levels. The relative abundance of Firmicutes (F) and Bacteroides (G) of three groups at the phylum levels. (I) The relative abundance of Bacteroides at genus levels. Data are means ± SD, *n* = 3, ^#^*P* < 0.05, ^###^*P* < 0.001 *vs.* the Control. ^*^P < 0.05, ^***^P < 0.001 *vs.* the Model. **Fig. S11** Metabolic characteristics of ApoE^-/-^ mice in Control, Model, and (Lipo+M)@E NPs groups. (A) Volcano plots show differential metabolites among groups in positive ion and negative ion modes. (B) PLS-DA score plot of the groups in two ion modes based on LC-MS technology. (C) Heatmaps show the differences in metabolites among the three groups. (D) Venn diagram compares overlap and unique differential metabolites among the groups. **Fig. S12** (Lipo+M)@E NPs reverse metabolic disorders caused by HFD. (A) KEGG pathway between Control, Model, and (Lipo+M)@E group. (*P* < 0.05). The levels of metabolites in different metabolic pathways including Chenodcoxycholic Acid (B), Taurochenodeoxycholic acid (C), Deoxycholic acid (D), Pantothenic acid (E), Ursodeoxycholic acid (F), and Palmitoylethanolamide (G) in Control, Model, and (Lipo+M)@E group. Data are means ± SD, n = 4, ^#^P < 0.05, ^##^P < 0.01, ^###^P < 0.001 *vs.* the Control. **P < 0.01, ***P < 0.001 *vs.* the Model. **Fig. S13** Effect of (Lipo+M)@E NPs on serum TC and TG levels in ApoE^-/-^ mice. Data are means ± SD, *n* = 5, ^###^*P* < 0.001 *vs.* the Control. ^***^P < 0.001 *vs.* the Model. **Fig. S14** Proposed mechanism of (Lipo+M)@E NPs in attenuating atherosclerosis. (Lipo+M)@E NPs alleviate atherosclerosis in vivo, due to reduce PCSK9, decrease LDLR degradation, while regulating gut microbiota, bile acids, and cholesterol metabolism. **Fig. S15** In vitro biocompatibility. (A) Hemolysis of RBCs at various concentrations of different materials. (B) The microscopy image of the hemolytic test, [Lipo]=3.6 μg/mL. Scale bars = 20 µm. (C) Platelet activation assay with different formulations. [Lipo]=3.6 μg/mL. (D) In vitro cytotoxicity evaluation of (Lipo+M)@E NPs. **Fig. S16** In vivo security assessment. (A) H&E-stained images of the heart, liver, spleen, lung, and kidney of ApoE^-/-^ mice with different treatments. Scale bars = 100 µm. (B&C) Blood routine and liver-kidney assays. *n* = 5.

## Data Availability

All data generated or analyzed during this study are included in this published article.

## References

[1] Bennett MR, Sinha S, Owens GK (2016). Vascular smooth muscle cells in atherosclerosis. Circ Res.

[2] Frismantiene A, Philippova M, Erne P, Resink TJ (2018). Smooth muscle cell-driven vascular diseases and molecular mechanisms of VSMC plasticity. Cell Signal.

[3] Chistiakov DA, Orekhov AN, Bobryshev YV (2015). Vascular smooth muscle cell in atherosclerosis. Acta Physiol (Oxf).

[4] Wirka RC, Wagh D, Paik DT, Pjanic M, Nguyen T, Miller CL (2019). Atheroprotective roles of smooth muscle cell phenotypic modulation and the TCF21 disease gene as revealed by single-cell analysis. Nat Med.

[5] Ji J, Feng M, Niu X, Zhang X, Wang Y (2021). Liraglutide blocks the proliferation, migration and phenotypic switching of Homocysteine (Hcy)-induced vascular smooth muscle cells (VSMCs) by suppressing proprotein convertase subtilisin kexin9 (PCSK9)/low-density lipoprotein receptor (LDLR). Bioengineered.

[6] Alberts MJ, Thompson PD (2020). PCSK9 (proprotein convertase subtilisin-kexin type 9) inhibition and stroke prevention: another step forward. Stroke.

[7] Maruf A, Wang Y, Yin T, Huang J, Wang N, Durkan C (2019). Atherosclerosis treatment with stimuli-responsive nanoagents: recent advances and future perspectives. Adv Healthc Mater.

[8] Nadimi AE, Ebrahimipour SY, Afshar EG, Falahati-Pour SK, Ahmadi Z, Mohammadinejad R (2018). Nano-scale drug delivery systems for antiarrhythmic agents. Eur J Med Chem.

[9] Harel-Adar T, Ben Mordechai T, Amsalem Y, Feinberg MS, Leor J, Cohen S (2011). Modulation of cardiac macrophages by phosphatidylserine-presenting liposomes improves infarct repair. Proc Natl Acad Sci USA.

[10] Milla P, Dosio F, Cattel L (2012). PEGylation of proteins and liposomes: a powerful and flexible strategy to improve the drug delivery. Curr Drug Metab.

[11] Anchordoquy TJ, Barenholz Y, Boraschi D, Chorny M, Decuzzi P, Dobrovolskaia MA (2017). Mechanisms and barriers in cancer nanomedicine: addressing challenges, Looking for Solutions. ACS Nano.

[12] Jiang Y, Krishnan N, Zhou J, Chekuri S, Wei X, Kroll AV (2020). Engineered cell-membrane-coated nanoparticles directly present tumor antigens to promote anticancer immunity. Adv Mater.

[13] Peng R, Ji H, Jin L, Lin S, Huang Y, Xu K (2020). Macrophage-based therapies for atherosclerosis management. J Immunol Res.

[14] ZareKazemabadi F, Heydarinasab A, Akbarzadeh A, Ardjmand M (2019). Preparation, characterization and in vitro evaluation of PEGylated nanoliposomal containing etoposide on lung cancer. Artif Cells Nanomed Biotechnol.

[15] Huo X, Wang Z, Xiao X, Yang C, Su J (2021). Oral administration of nanopeptide CMCS-20H conspicuously boosts immunity and precautionary effect against bacterial infection in fish. Front Immunol.

[16] Liu B, Wang W, Fan J, Long Y, Xiao F, Daniyal M (2019). RBC membrane camouflaged prussian blue nanoparticles for gamabutolin loading and combined chemo/photothermal therapy of breast cancer. Biomaterials.

[17] Zhou H, You P, Liu H, Fan J, Tong C, Yang A (2022). Artemisinin and Procyanidins loaded multifunctional nanocomplexes alleviate atherosclerosis via simultaneously modulating lipid influx and cholesterol efflux. J Control Release.

[18] Xiao C, Tong C, Fan J, Wang Z, Xie Q, Long Y (2021). Biomimetic nanoparticles loading with gamabutolin-indomethacin for chemo/photothermal therapy of cervical cancer and anti-inflammation. J Control Release.

[19] Liu B, Yan W, Luo L, Wu S, Wang Y, Zhong Y (2021). Macrophage membrane camouflaged reactive oxygen species responsive nanomedicine for efficiently inhibiting the vascular intimal hyperplasia. J Nanobiotechnol.

[20] Gregory JL, Morand EF, McKeown SJ, Ralph JA, Hall P, Yang YH (2006). Macrophage migration inhibitory factor induces macrophage recruitment via CC chemokine ligand 2. J Immunol.

[21] Hart R, Greaves DR (2010). Chemerin contributes to inflammation by promoting macrophage adhesion to VCAM-1 and fibronectin through clustering of VLA-4 and VLA-5. J Immunol.

[22] Smith CW, Marlin SD, Rothlein R, Toman C, Anderson DC (1989). Cooperative interactions of LFA-1 and Mac-1 with intercellular adhesion molecule-1 in facilitating adherence and transendothelial migration of human neutrophils in vitro. J Clin Invest.

[23] He W, Xing X, Wang X, Wu D, Mitragotri S (2020). Nanocarrier mediated cytosolic delivery of biopharmaceuticals. Adv Func Mater.

[24] Hu CM, Fang RH, Luk BT, Zhang L (2014). Polymeric nanotherapeutics: clinical development and advances in stealth functionalization strategies. Nanoscale.

[25] Han Y, Pan H, Li W, Chen Z, Ma A, Yin T (2019). T cell membrane mimicking nanoparticles with bioorthogonal targeting and immune recognition for enhanced photothermal therapy. Adv Sci (Weinh).

[26] Long Y, Wang Z, Fan J, Yuan L, Tong C, Zhao Y (2021). A hybrid membrane coating nanodrug system against gastric cancer via the VEGFR2/STAT3 signaling pathway. J Mater Chem B.

[27] Xie Q, Liu Y, Long Y, Wang Z, Jiang S, Ahmed R (2021). Hybrid-cell membrane-coated nanocomplex-loaded chikusetsusaponin IVa methyl ester for a combinational therapy against breast cancer assisted by Ce6. Biomater Sci.

[28] Li R, He Y, Zhu Y, Jiang L, Zhang S, Qin J (2019). Route to rheumatoid arthritis by macrophage-derived microvesicle-coated nanoparticles. Nano Lett.

[29] Ferri N, Corsini A, Macchi C, Magni P, Ruscica M (2016). Proprotein convertase subtilisin kexin type 9 and high-density lipoprotein metabolism: experimental animal models and clinical evidence. Transl Res.

[30] Paudel KR, Karki R, Kim DW (2016). Cepharanthine inhibits in vitro VSMC proliferation and migration and vascular inflammatory responses mediated by RAW264.7. Toxicol In Vitro..

[31] Zhang M, Urabe G, Little C, Wang B, Kent AM, Huang Y (2018). HDAC6 regulates the MRTF-A/SRF axis and vascular smooth muscle cell plasticity. JACC Basic Transl Sci.

[32] Liu X, Luo F, Pan K, Wu W, Chen H (2007). High glucose upregulates connective tissue growth factor expression in human vascular smooth muscle cells. BMC Cell Biol.

[33] Nürnberg A, Kitzing T, Grosse R (2011). Nucleating actin for invasion. Nat Rev Cancer.

[34] Sanz-Moreno V, Marshall CJ (2010). The plasticity of cytoskeletal dynamics underlying neoplastic cell migration. Curr Opin Cell Biol.

[35] Khan R, Agrotis A, Bobik A (2007). Understanding the role of transforming growth factor-beta1 in intimal thickening after vascular injury. Cardiovasc Res.

[36] Dubland JA, Francis GA (2016). So much cholesterol: the unrecognized importance of smooth muscle cells in atherosclerotic foam cell formation. Curr Opin Lipidol.

[37] Allahverdian S, Chehroudi AC, McManus BM, Abraham T, Francis GA (2014). Contribution of intimal smooth muscle cells to cholesterol accumulation and macrophage-like cells in human atherosclerosis. Circulation.

[38] Sha X, Dai Y, Chong L, Wei M, Xing M, Zhang C (2022). Pro-efferocytic macrophage membrane biomimetic nanoparticles for the synergistic treatment of atherosclerosis via competition effect. J Nanobiotechnol.

[39] Liang H, Huang K, Su T, Li Z, Hu S, Dinh PU (2018). Mesenchymal stem cell/red blood cell-inspired nanoparticle therapy in mice with carbon tetrachloride-induced acute liver failure. ACS Nano.

[40] Bäck M, Yurdagul A, Tabas I, Öörni K, Kovanen PT (2019). Inflammation and its resolution in atherosclerosis: mediators and therapeutic opportunities. Nat Rev Cardiol.

[41] Xu S, Ilyas I, Little PJ, Li H, Kamato D, Zheng X (2021). Endothelial dysfunction in atherosclerotic cardiovascular diseases and beyond: from mechanism to pharmacotherapies. Pharmacol Rev.

[42] Durham AL, Speer MY, Scatena M, Giachelli CM, Shanahan CM (2018). Role of smooth muscle cells in vascular calcification: implications in atherosclerosis and arterial stiffness. Cardiovasc Res.

[43] Silvestre-Roig C, de Winther MP, Weber C, Daemen MJ, Lutgens E, Soehnlein O (2014). Atherosclerotic plaque destabilization: mechanisms, models, and therapeutic strategies. Circ Res.

[44] Drosos I, Tavridou A, Kolios G (2015). New aspects on the metabolic role of intestinal microbiota in the development of atherosclerosis. Metabolism.

[45] Org E, Mehrabian M, Lusis AJ (2015). Unraveling the environmental and genetic interactions in atherosclerosis: central role of the gut microbiota. Atherosclerosis.

[46] Emoto T, Yamashita T, Sasaki N, Hirota Y, Hayashi T, So A (2016). Analysis of gut microbiota in coronary artery disease patients: a possible link between gut microbiota and coronary artery disease. J Atheroscler Thromb.

[47] Lyu M, Wang YF, Fan GW, Wang XY, Xu SY, Zhu Y (2017). Balancing herbal medicine and functional food for prevention and treatment of cardiometabolic diseases through modulating gut microbiota. Front Microbiol.

[48] Gibiino G, Lopetuso LR, Scaldaferri F, Rizzatti G, Binda C, Gasbarrini A (2018). Exploring bacteroidetes: metabolic key points and immunological tricks of our gut commensals. Dig Liver Dis.

[49] Mu H, Zhou Q, Yang R, Zeng J, Li X, Zhang R (2020). Naringin attenuates high fat diet induced non-alcoholic fatty liver disease and gut bacterial dysbiosis in mice. Front Microbiol.

[50] Liu Y, Gao Y, Ma F, Sun M, Mu G, Tuo Y (2020). The ameliorative effect of *Lactobacillus*
*plantarum* Y44 oral administration on inflammation and lipid metabolism in obese mice fed with a high fat diet. Food Funct.

[51] Iida M, Harada S, Takebayashi T (2019). Application of metabolomics to epidemiological studies of atherosclerosis and cardiovascular disease. J Atheroscler Thromb.

[52] Simental-Mendía LE, Simental-Mendía M, Sánchez-García A, Banach M, Serban MC, Cicero AFG (2019). Impact of ursodeoxycholic acid on circulating lipid concentrations: a systematic review and meta-analysis of randomized placebo-controlled trials. Lipids Health Dis.

[53] Gugliandolo E, Fusco R, Biundo F, D'Amico R, Benedetto F, Di Paola R (2017). Palmitoylethanolamide and Polydatin combination reduces inflammation and oxidative stress in vascular injury. Pharmacol Res.

