# Supplementary material for: Evolocumab loaded Bio-Liposomes for efficient atherosclerosis therapy
Source: J Nanobiotechnology. 2023 May 19;21:158. doi: 10.1186/s12951-023-01904-4 (PMC10199622; doi:10.1186/s12951-023-01904-4)
Supplement: Supplementary file 1 — Additional file 1. Fig. S1 Phagocytosis of Rho in VSMCs and HUVECs in a transwell. BF indicates bright field. Fig. S2 Cell uptake mechanism of M@Lipo NPs. CLSM image (A) and quantitation (B) of the VSMCs uptake for M@Lipo NPs in different inhibitor groups. Scale bar = 60 μm. Data are means ± SD, n = 3, *P < 0.05, **P < 0.01, ***P < 0.001 vs. the Control. Fig. S3 Immune-escape properties of (Lipo+M)@E NPs in vitro. Confocal images (A) and mean fluorescence intensity (MFI) (B) of different concentrations of Lipo NPs and M@Lipo NPs phagocytosed by RAW264.7 cells. Scale bars = 60 μm. Data are means ± SD, n = 3, *P < 0.05, **P < 0.01, ***P < 0.001 vs. the Lipo. Fig. S4 Distribution of M@Lipo NPs in major organs of ApoE-/- mice. (A) Fluorescence imaging of the major organs of ApoE-/- mice with different treatments for 12 h. (B) The relative fluorescence signal of major organs (n = 3). Statistically significant differences between M@Lipo@Ce6 NPs in different organs and in the livers (###P < 0.001); statistically significant differences between Lipo@Ce6 NPs and M@Lipo@Ce6 NPs in the livers (*P < 0.05). Fig. S5 LPS can increase the expression of PCSK9 in VSMCs. LPS-induced expression of PCSK9 in VSMCs in a dose-dependent fashion (measured by western blot). Data are means ± SD, n = 3, #P < 0.05, ##P < 0.01 vs. the Control. Fig. S6 Evol can reduce the expression of PCSK9 in VSMCs. Western blot assay of the levels of PCSK9 in VSMCs treated with different concentrations of Evol (2.5 nM, 5.0 nM, and 10.0 nM). Data are means ± SD, n = 3, ###P < 0.001 vs. the Control. **P < 0.01, ***P < 0.001 vs. the Model. Fig. S7 Transcriptomic analysis of the Model and (Lipo+M)@E group. (A) Volcano plots show differential expression genes between the Model and (Lipo+M)@E NPs group. Red and blue represent genes upregulation and downregulation, respectively. Biological process (B), Molecular function (C), and Cellular component (D) in GO function of the Model and (Lipo+M)@E NPs group. (E) GSEA enr [file 12951_2023_1904_MOESM1_ESM.docx]

**Evolocumab loaded Bio-Liposomes for efficient atherosclerosis therapy**

Zhenxian Li^a#^, Haimei Zhu^e#^, Hao Liu^c^, Dayue Liu^d^, Jianhe Liu^a^, Jiazheng Jiang^a^, Yi Zhang^a^, Zhang Qin^a^, Yijia Xu^a^, Yuan Peng^a^, Bin Liu^b,d⁎^, Yun Long^a⁎^

*^a^ Department of Cardiology, The First Hospital of Hunan University of Chinese Medicine, Branch of National Clinical Research Center for Chinese Medicine Cardiology, Changsha 410007, China*

*^b^ College of Biology, Hunan University, Changsha 410082, China*

^c^ *Department of Rehabilitation, The Second Xiangya Hospital, Central South University, Changsha 410011, China*

*^d^ NHC Key Laboratory of Metabolic Cardiovascular Diseases Research, Department of Physiology and Pathophysiology, School of Basic Medical Sciences, Ningxia Medical University, Yinchuan 750004, China*

*^e^ Department of Pain, The First Hospital of Hunan University of Chinese Medicine, Changsha 410007, China*

⁎ *Corresponding authors at:*

*Yun Long, Ph.D*

*The First Hospital of Hunan University of Chinese Medicine, Changsha 410007, China.*

*Bin Liu, Ph.D*

*College of Biology, Hunan University, Changsha 410082, China*

*E-mail addresses:* wwlyf@126.com (Y. Long); binliu2001@hotmail.com (B. Liu).

^#^ These authors contributed equally to this work and should be regarded as the first authors.

## Fig. S1


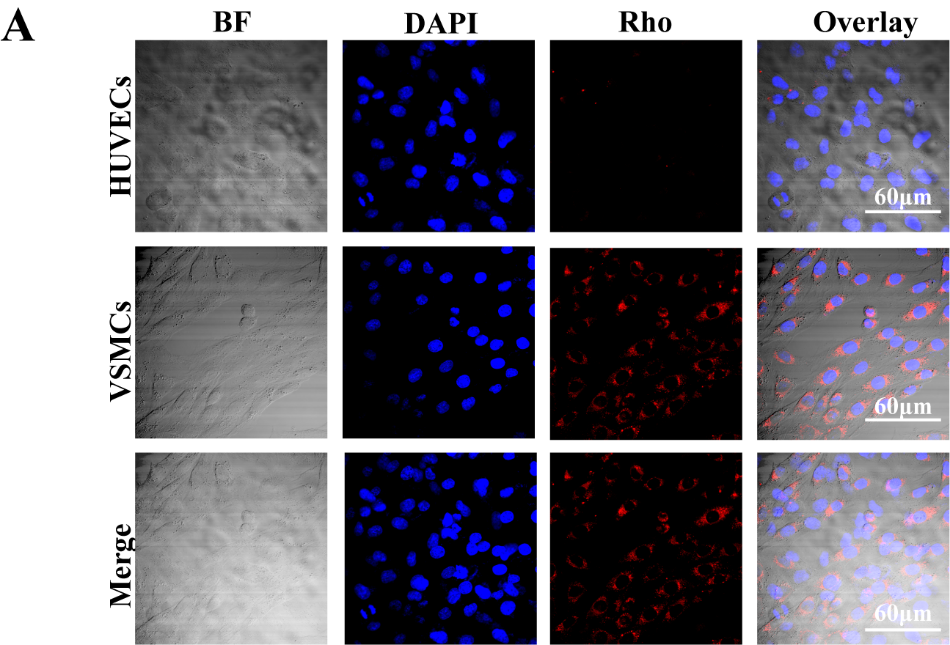


**Fig. S1** Phagocytosis of Rho in VSMCs and HUVECs in a transwell. BF indicates bright field.

## Fig. S2


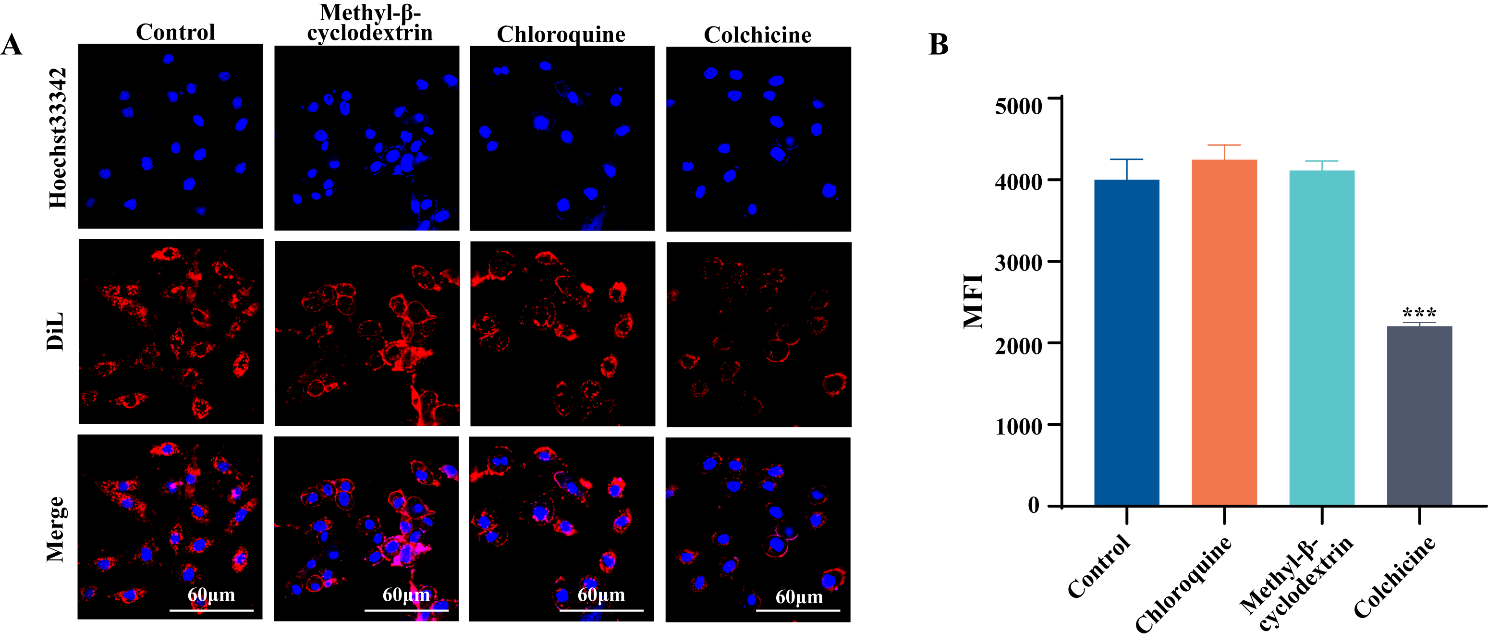


**Fig. S2** Cell uptake mechanism of M@Lipo NPs. CLSM image (A) and quantitation (B) of the VSMCs uptake for M@Lipo NPs in different inhibitor groups. Scale bar = 60 μm. Data are means ± SD, *n* = 3, ^*^*P* < 0.05, ^**^*P* < 0.01, ^***^*P* < 0.001 *vs.* the Control.

## Fig. S3


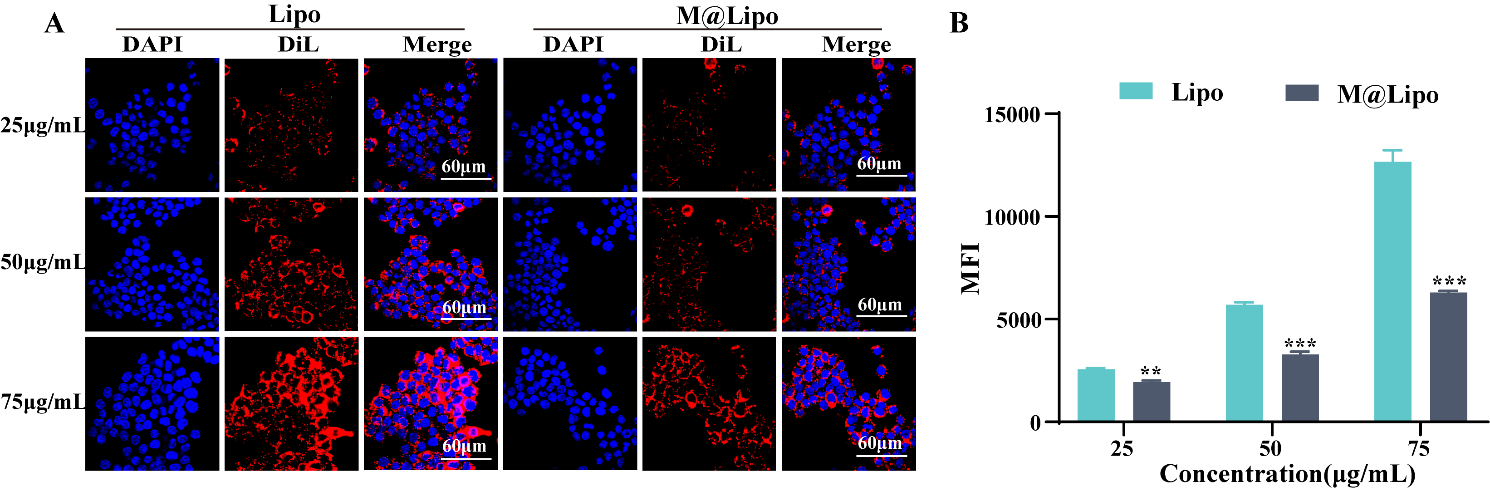


**Fig. S3** Immune-escape properties of (Lipo+M)@E NPs *in* *vitro*. Confocal images (A) and mean fluorescence intensity (MFI) (B) of different concentrations of Lipo NPs and M@Lipo NPs phagocytosed by RAW264.7 cells. Scale bars = 60 μm. Data are means ± SD, *n* = 3, **P* < 0.05, ***P* < 0.01, ****P* < 0.001 *vs*. the Lipo.

## Fig. S4


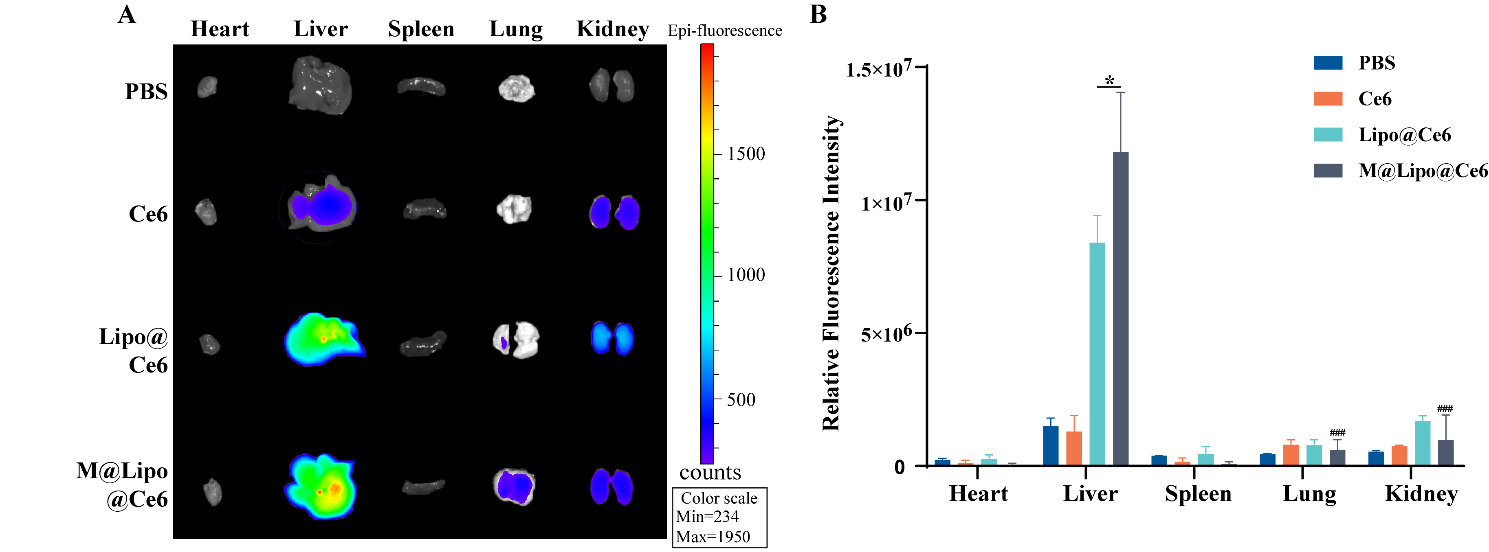


**Fig. S4** Distribution of M@Lipo NPs in major organs of ApoE^-/-^ mice. (A) Fluorescence imaging of the major organs of ApoE^-/-^ mice with different treatments for 12 h. (B) The relative fluorescence signal of major organs (*n* = 3). Statistically significant differences between M@Lipo@Ce6 NPs in different organs and in the livers (^###^*P* < 0.001); statistically significant differences between Lipo@Ce6 NPs and M@Lipo@Ce6 NPs in the livers (^*^*P* < 0.05).

## Fig. S5


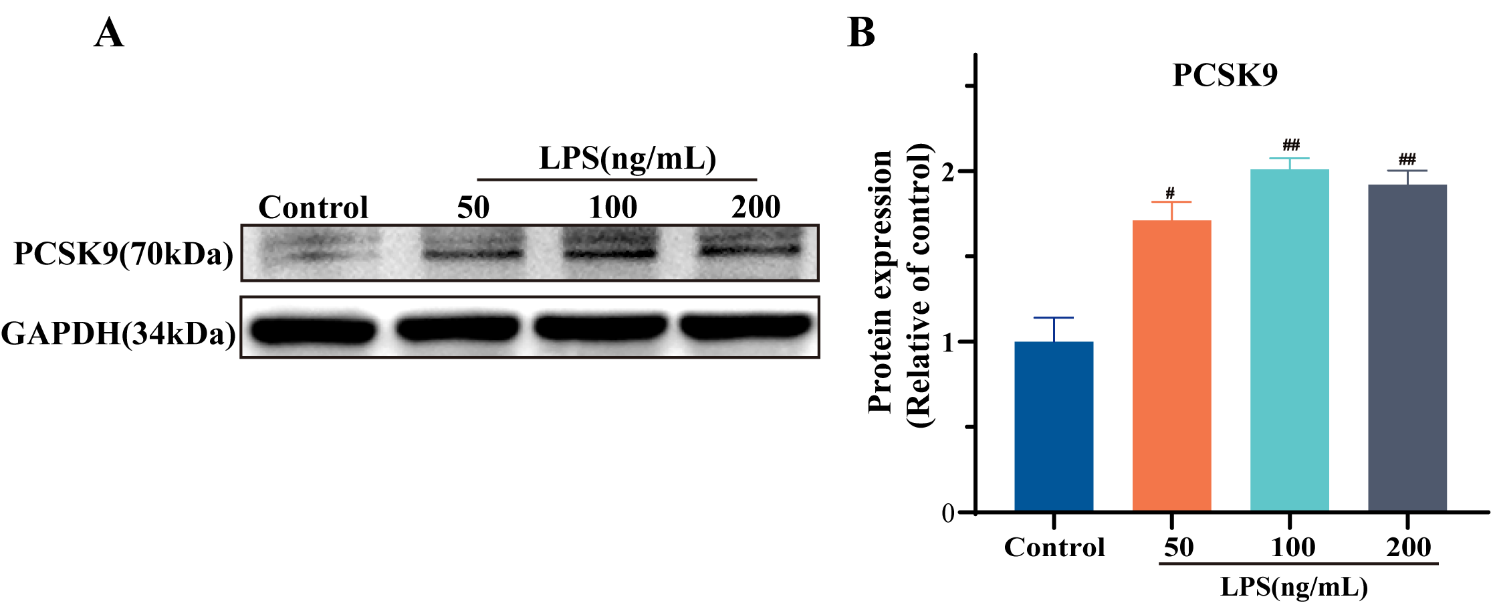


**Fig. S5** LPS can increase the expression of PCSK9 in VSMCs. LPS-induced expression of PCSK9 in VSMCs in a dose-dependent fashion (measured by western blot). Data are means ± SD, *n* = 3, **^#^***P* < 0.05, **^##^***P* < 0.01 *vs.* the Control.

## Fig. S6


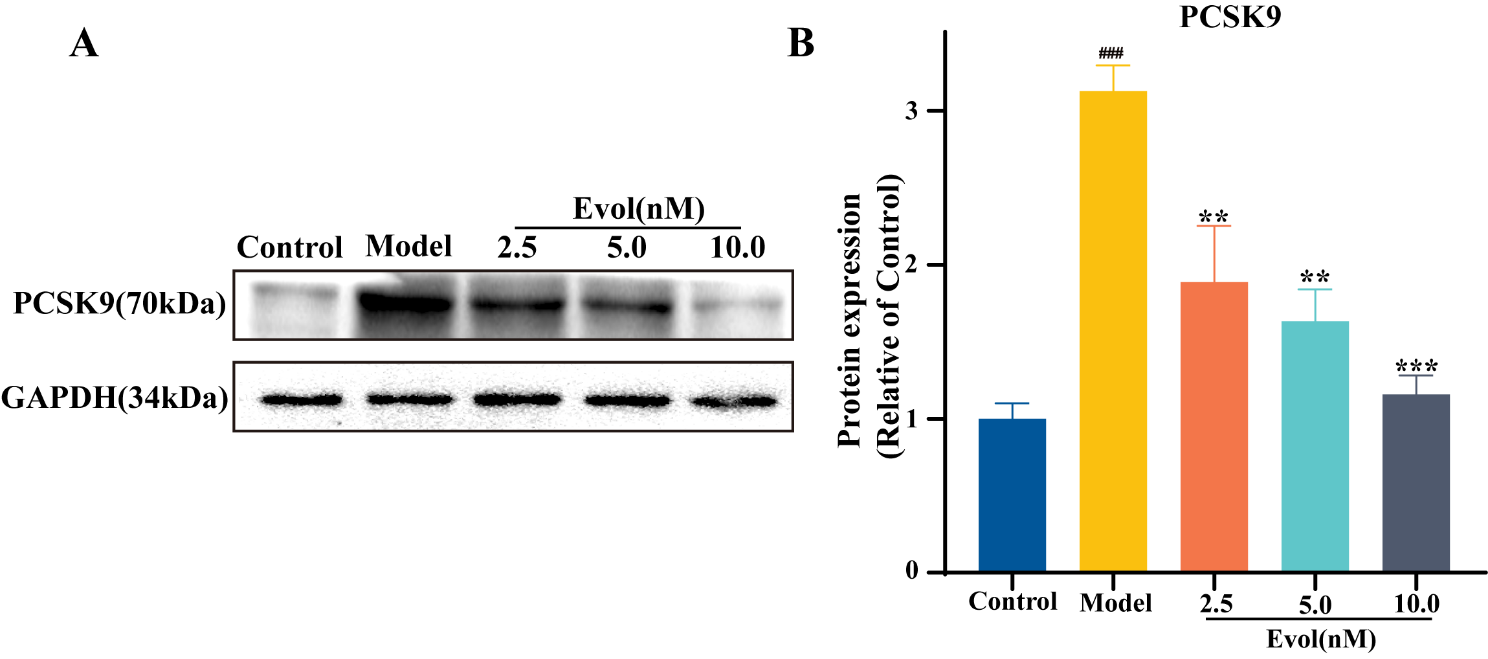


**Fig. S6** Evol can reduce the expression of PCSK9 in VSMCs. Western blot assay of the levels of PCSK9 in VSMCs treated with different concentrations of Evol (2.5 nM, 5.0 nM, and 10.0 nM). Data are means ± SD, *n* = 3, **^###^***P* < 0.001 *vs.* the Control. ^**^*P* < 0.01, ^***^*P* < 0.001 *vs.* the Model.

## Fig. S7


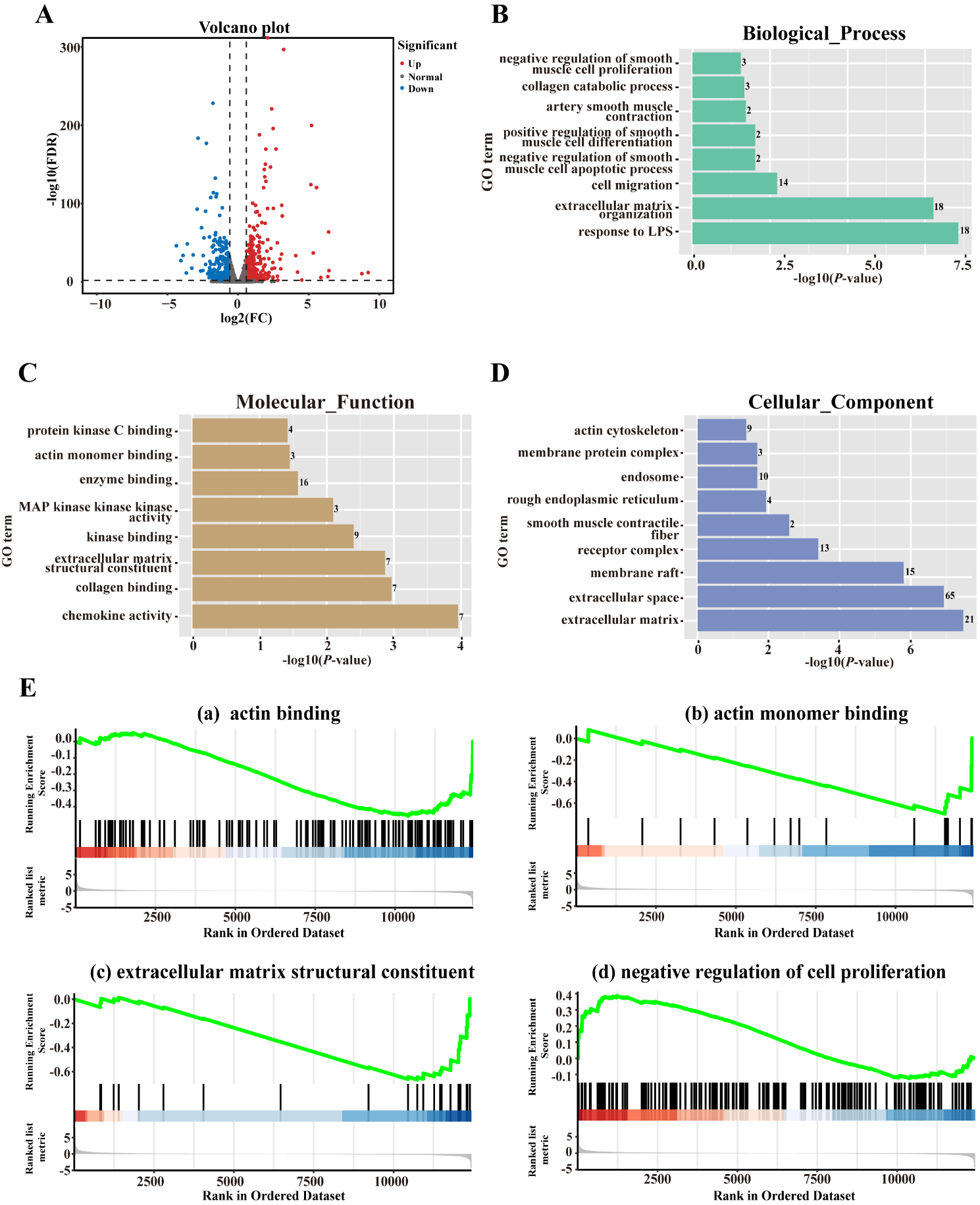


**Fig. S7** Transcriptomic analysis of the Model and (Lipo+M)@E group. (A) Volcano plots show differential expression gene between the Model and (Lipo+M)@E NPs group. Red and blue represent genes upregulation and downregulation, respectively. Biological process (B), Molecular function (C), and Cellular component (D) in GO function of the Model and (Lipo+M)@E NPs group. (E) GSEA enrichment plots of differentially expressed genes in the Model and (Lipo+M)@E group. (a) actin binding. (b) actin monomer binding. (c) extracellular matrix structural constituent. (d) negative regulation of cell proliferation. *P* < 0.05.

## Fig. S8


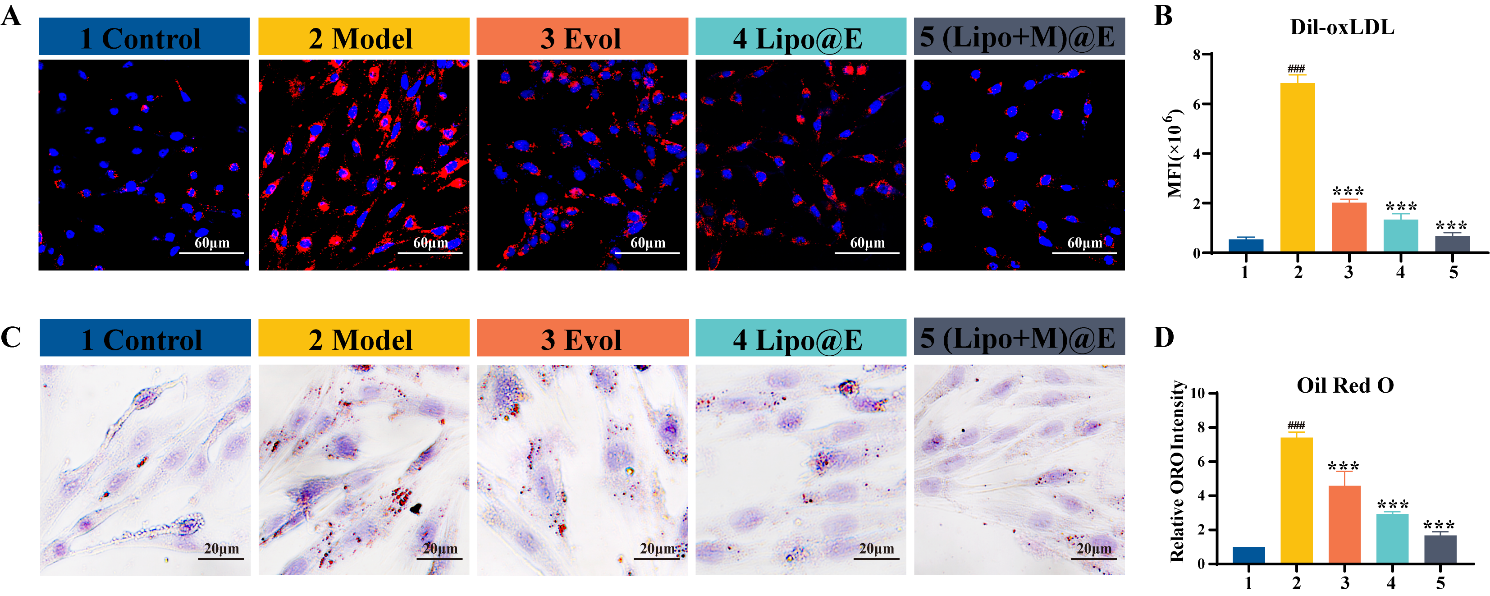


**Fig. S8** Cellular uptake of oxLDL and Oil Red O staining of VSMCs. Representative fluorescence images (A) and quantification (B) of DiL-oxLDL uptake in VSMCs after incubation for 4 h. Scale bars = 60 µm. Images (C) and quantification (D) of oxLDL internalization in VSMCs. Scale bars = 20 µm. Data are means ± SD, *n* = 3, ^###^*P* < 0.001 *vs.* the Control. ^***^*P* < 0.001 *vs.* the Model.

## Fig. S9


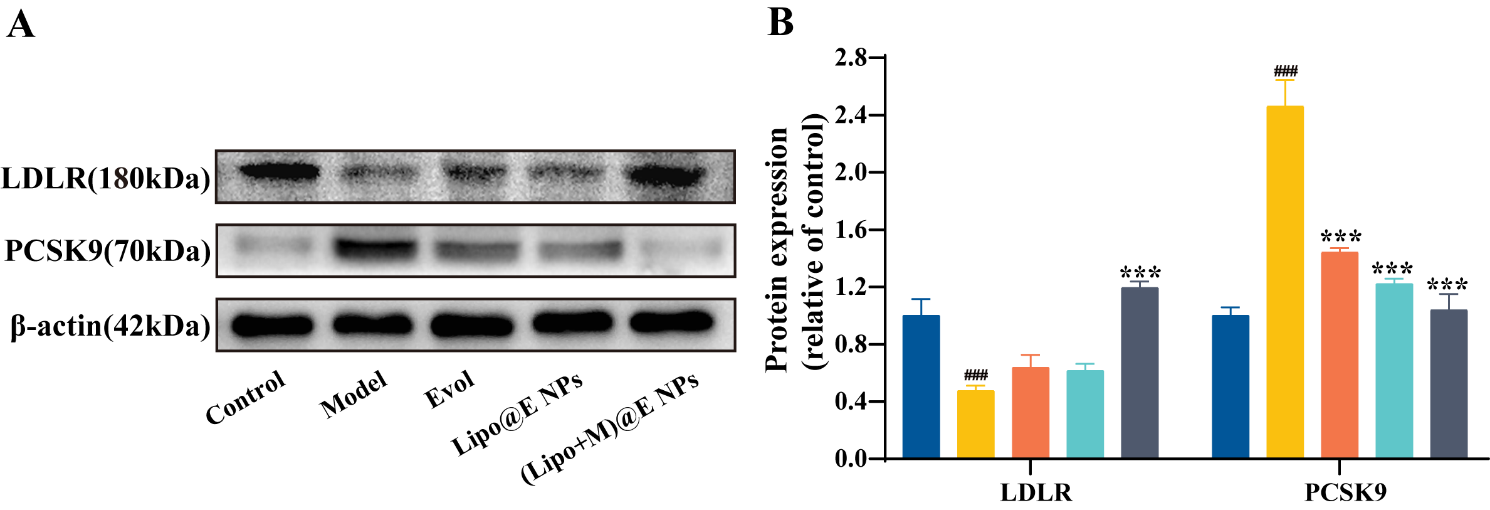


**Fig. S9.** (Lipo+M)@E NPs regulated LDLR and PCSK9 levels in livers of ApoE^-/-^ mice. **(**A) Represented photograph showing the protein expressions of LDLR and PCSK9 in livers of ApoE^-/-^ mice after treatment with Evol, Lipo@E NPs, and (Lipo+M)@E NPs. (B) The relative quantification analysis of LDLR and PCSK9. Data are means ± SD, *n* = 3, ^###^*P* < 0.001 *vs.* the Control. ^***^*P* < 0.001 *vs.* the Model.

## Fig. S10


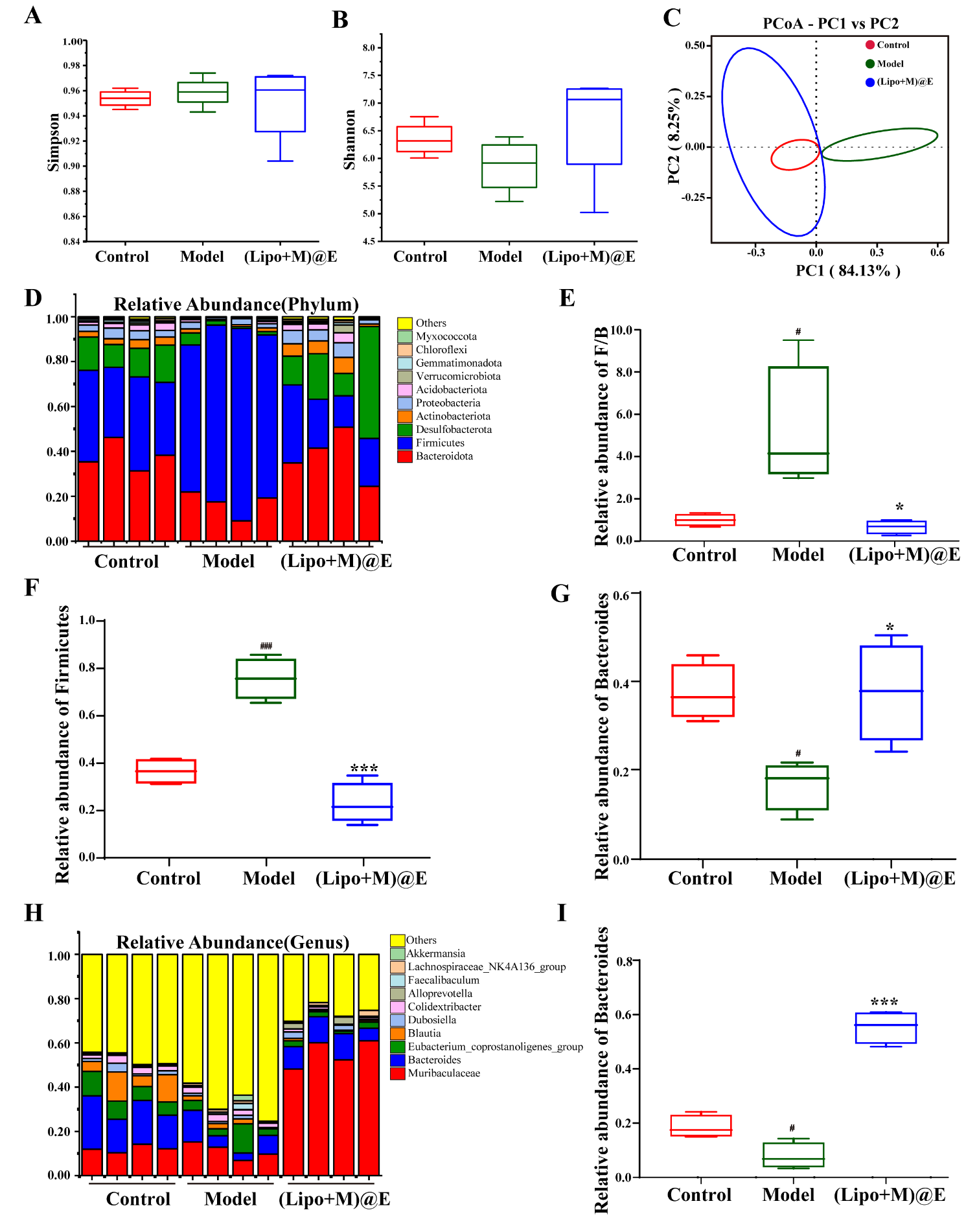


**Fig. S10.** (Lipo+M)@E NPs can alter the composition of intestinal flora in ApoE^-/-^ atherosclerosis mice. (A&B) Shannon indexes and Simpson indexes of the Control, Model, and (Lipo+M)@E NPs group. (C) PCoA analysis of the Control, Model, and (Lipo+M)@E NPs group. The relative abundance of gut microbiota at phylum levels (D) and genus levels (H) in the Control, Model, and (Lipo+M)@E NPs groups. (E) The F to B ratio of three groups at the phylum levels. The relative abundance of Firmicutes (F) and Bacteroides (G) of three groups at the phylum levels. (I) The relative abundance of Bacteroides at genus levels. Data are means ± SD, *n* = 3, ^#^*P* < 0.05, ^###^*P* < 0.001 *vs.* the Control. ^*^*P* < 0.05, ^***^*P* < 0.001 *vs.* the Model.

## Fig. S11


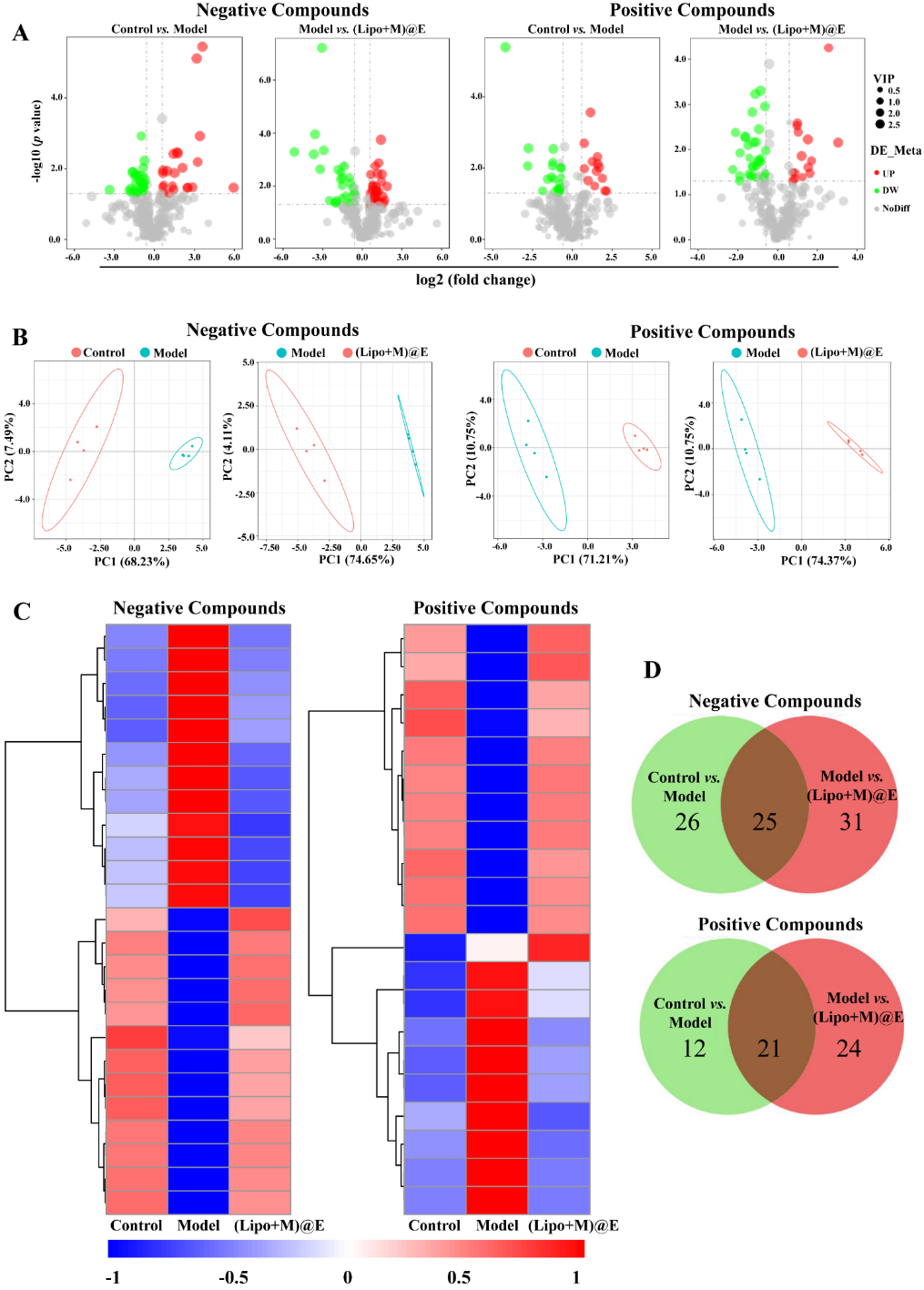


**Fig. S11** Metabolic characteristics of ApoE^-/-^ mice in Control, Model, and (Lipo+M)@E NPs groups. (A) Volcano plots show differential metabolites among groups in positive ion and negative ion mode. (B) PLS-DA score plot of the groups in two ion modes based on LC-MS technology. (C) Heatmaps show the differences in metabolites among the three groups. (D) Venn diagram compares overlap and unique differential metabolites among the groups.

## Fig. S12


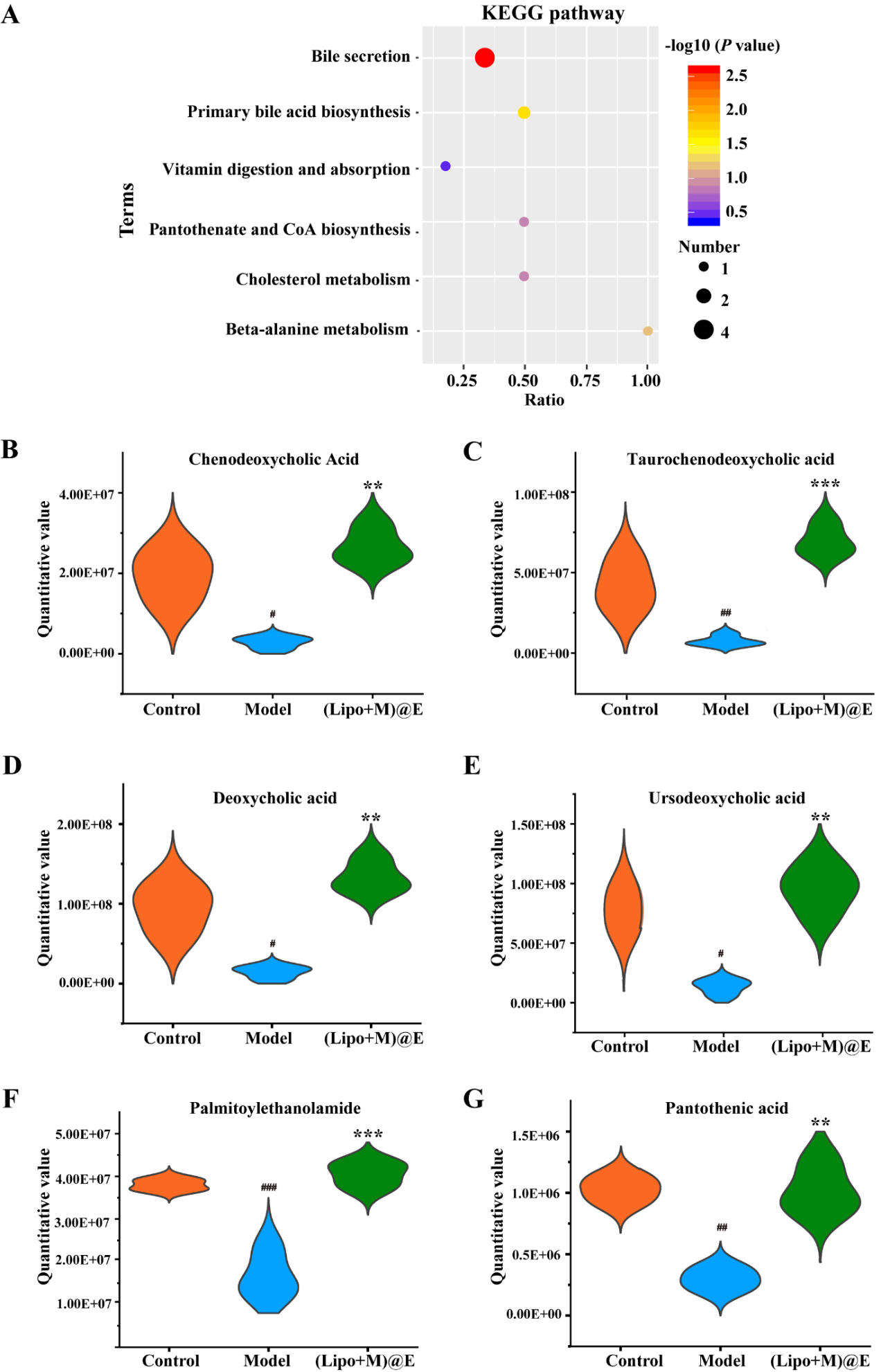


**Fig. S12** (Lipo+M)@E NPs reverse metabolic disorders caused by HFD. (A) KEGG pathway between Control, Model, and (Lipo+M)@E group. (*P* < 0.05). The levels of metabolites in different metabolic pathways including Chenodcoxycholic Acid (B), Taurochenodeoxycholic acid (C), Deoxycholic acid (D), Pantothenic acid (E), Ursodeoxycholic acid (F) and Palmitoylethanolamide (G) in Control, Model, and (Lipo+M)@E group. Data are means ± SD, *n* = 4, ^#^P < 0.05, ^##^P < 0.01, ^###^P < 0.001 *vs.* the Control. **P < 0.01, ***P < 0.001 *vs.* the Model.

## Fig. S13


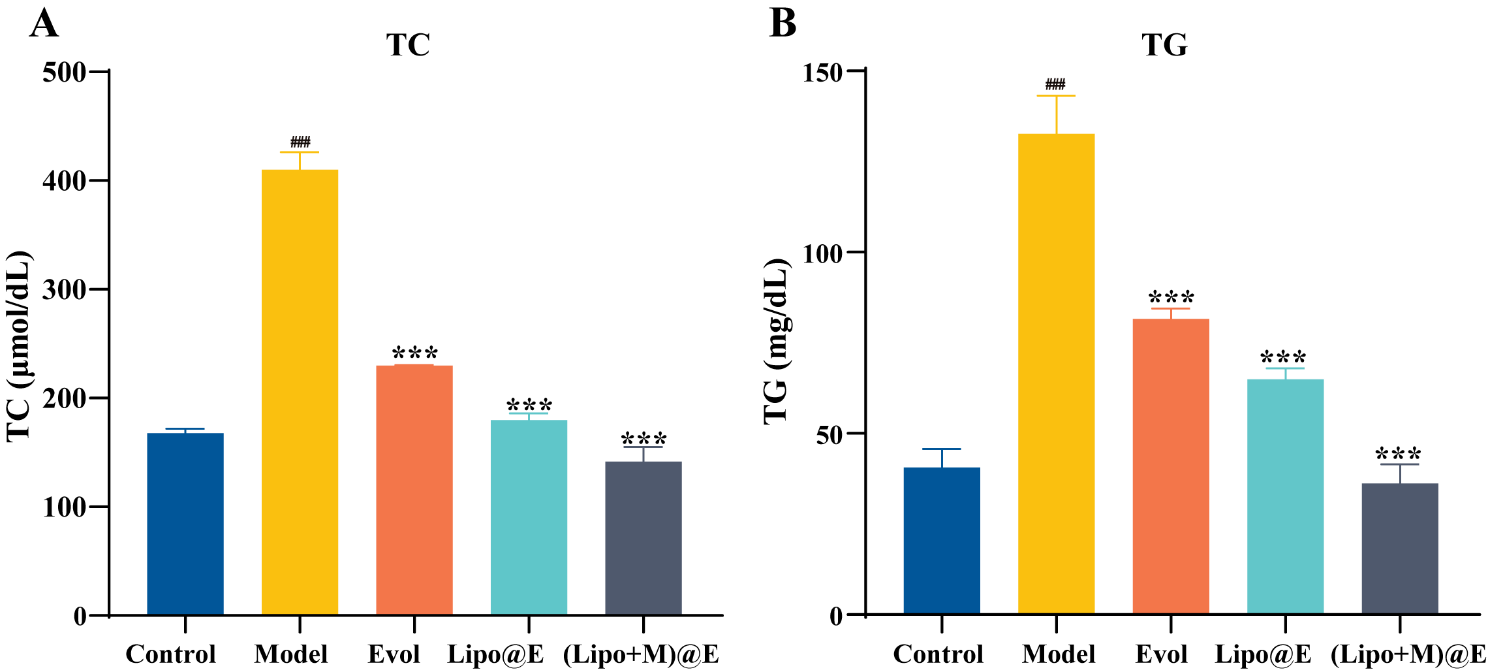


**Fig. S13** Effect of (Lipo+M)@E NPs on serum TC and TG levels in ApoE^-/-^ mice. Data are means ± SD, *n* = 5, ^###^*P* < 0.001 *vs.* the Control. ^***^*P* < 0.001 *vs.* the Model.

## Fig. S14


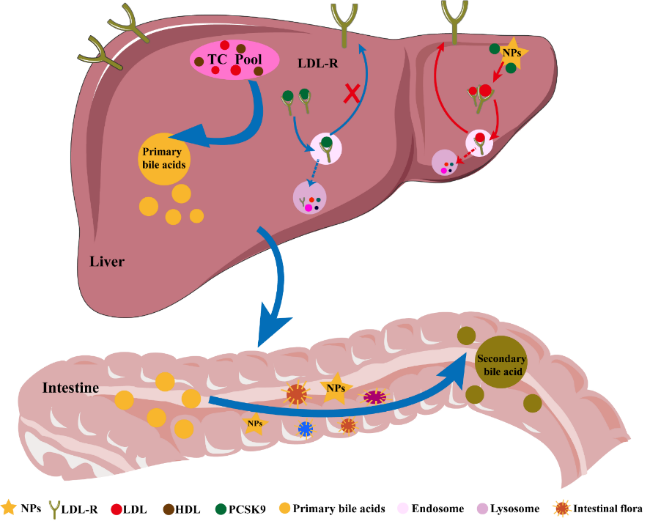


**Fig. S14** Proposed mechanism of (Lipo+M)@E NPs in attenuating atherosclerosis. (Lipo+M)@E NPs alleviate atherosclerosis *in vivo*, due to reduce PCSK9, decrease LDLR degradation, while regulating gut microbiota, bile acids and cholesterol metabolism.

## Fig. S15


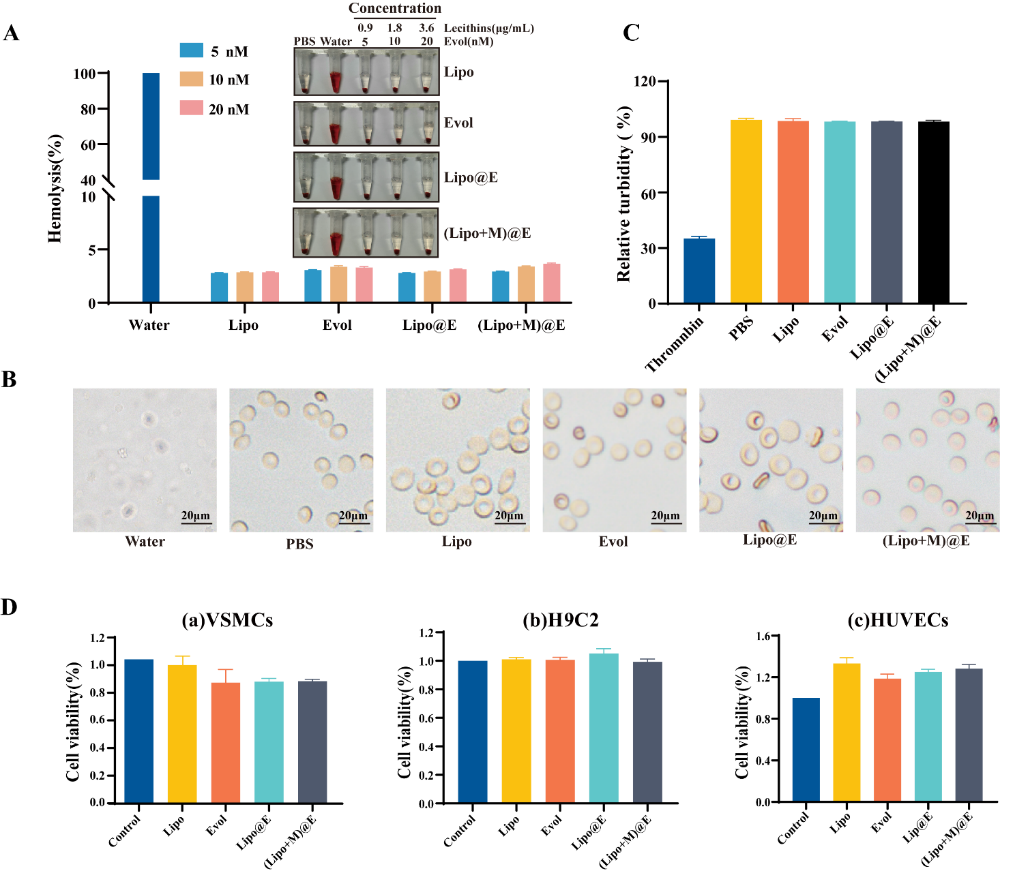


**Fig. S15** In *vitro* biocompatibility. (A) Hemolysis of RBCs at various concentrations of different materials. (B) The microscopy image of the hemolytic test, [Lipo]=3.6 μg/mL. Scale bars = 20 µm. (C) Platelet activation assay with different formulations. [Lipo]=3.6 μg/mL. (D) *In* *vitro* cytotoxicity evaluation of (Lipo+M)@E NPs.

## Fig. S16


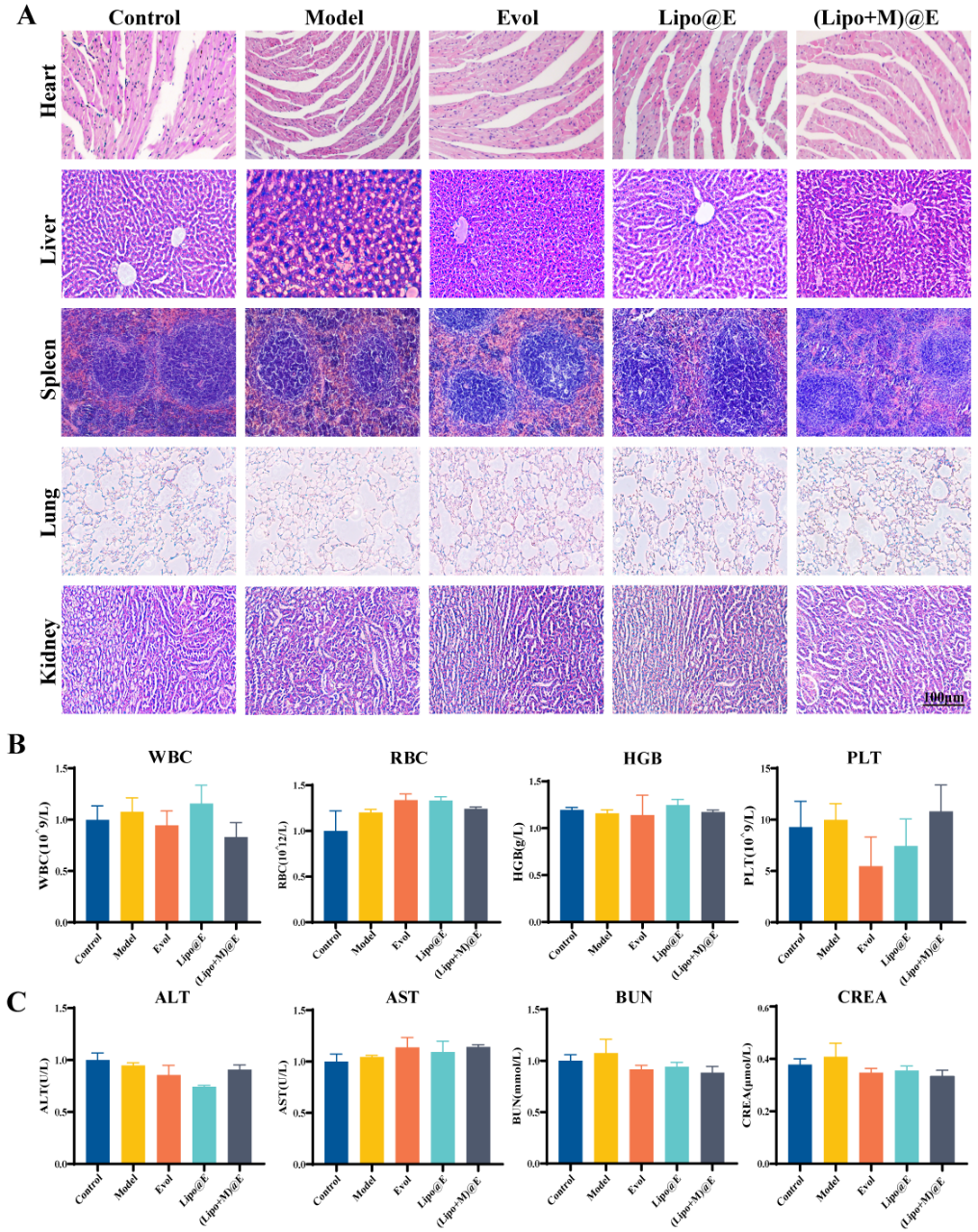


**Fig. S16** *In* *vivo* security assessment. (A) H&E-stained images of the heart, liver, spleen, lung, and kidney of ApoE^-/-^ mice with different treatments. Scale bars = 100 µm. (B&C) Blood routine and liver-kidney assays. *n* = 5.
